# Data on the verification and validation of segmentation and registration methods for diffusion MRI

**DOI:** 10.1016/j.dib.2016.06.049

**Published:** 2016-07-02

**Authors:** Oscar Esteban, Dominique Zosso, Alessandro Daducci, Meritxell Bach-Cuadra, María J. Ledesma-Carbayo, Jean-Philippe Thiran, Andres Santos

**Affiliations:** a Biomedical Image Technologies (BIT), ETSI Telecomunicación, Universidad Politécnica de Madrid, Madrid, Spain; b Centro de Investigación Biomédica en Red en Bioingeniería, Biomateriales y Nanomedicina (CIBER-BBN), Spain; c Department of Mathematics, University of California, Los Angeles (UCLA), Los Angeles, CA, USA; d Signal Processing Laboratory (LTS5), École Polytechnique Fédérale de Lausanne (EPFL), Lausanne, Switzerland; e Department of Radiology, CIBM, University Hospital Center (CHUV) and University of Lausanne (UNIL), Lausanne, Switzerland

**Keywords:** Neuroimage, Image processing, MRI methods, Diffusion MRI

## Abstract

The verification and validation of segmentation and registration methods is a necessary assessment in the development of new processing methods. However, verification and validation of diffusion MRI (dMRI) processing methods is challenging for the lack of gold-standard data. The data described here are related to the research article entitled “Surface-driven registration method for the structure-informed segmentation of diffusion MR images” [1], in which publicly available data are used to derive *golden*-standard reference-data to validate and evaluate segmentation and registration methods in dMRI.

**Specifications Table**

**Table:** 

Subject area	*Neuroimaging*
More specific subject area	*Image processing: registration and segmentation*
Type of data	*Figures, graphs and text*
How data was acquired	*In silico analysis of digital phantoms and real images from the Human Connectome Project*[2]*datasets*
Data format	*Analyzed data*
Experimental factors	*The FA (fractional anisotropy) and ADC (apparent diffusion coefficient) maps derived from the dMRI datasets, 3D triangular meshes computed from the T1-weighted MRI images, fieldmap images.*
Experimental features	*Residual alignment errors after image registration*
Data source location	*Spain*
Data accessibility	*Data is within this article and available online at* http://dx.doi.org/10.6084/m9.figshare.1397502

**Value of the data**

• Digital phantoms for the verification and validation of image processing methods. We release the workflows to generate the “gyrus”, “box”, “ball” and “L” phantoms, with the simulation of T1-weighted and T2-weighted contrasts.
• Flowcharts describing the workflows used to generate the random & synthetic distortions on the phantoms, as well as the theory-based warpings for real datasets, are also available. These items are useful in validation and benchmarking of image registration methods.
• All the software instrumentation is open-source and available in Github^1^ all the necessary workflows to reproduce our work in particular, and to create evaluation workflows in general are available.
• Reporting tools: sample reports of our evaluation framework are provided, facilitating the production of such information in further studies.

## 1 Data

**Fig. 1:**
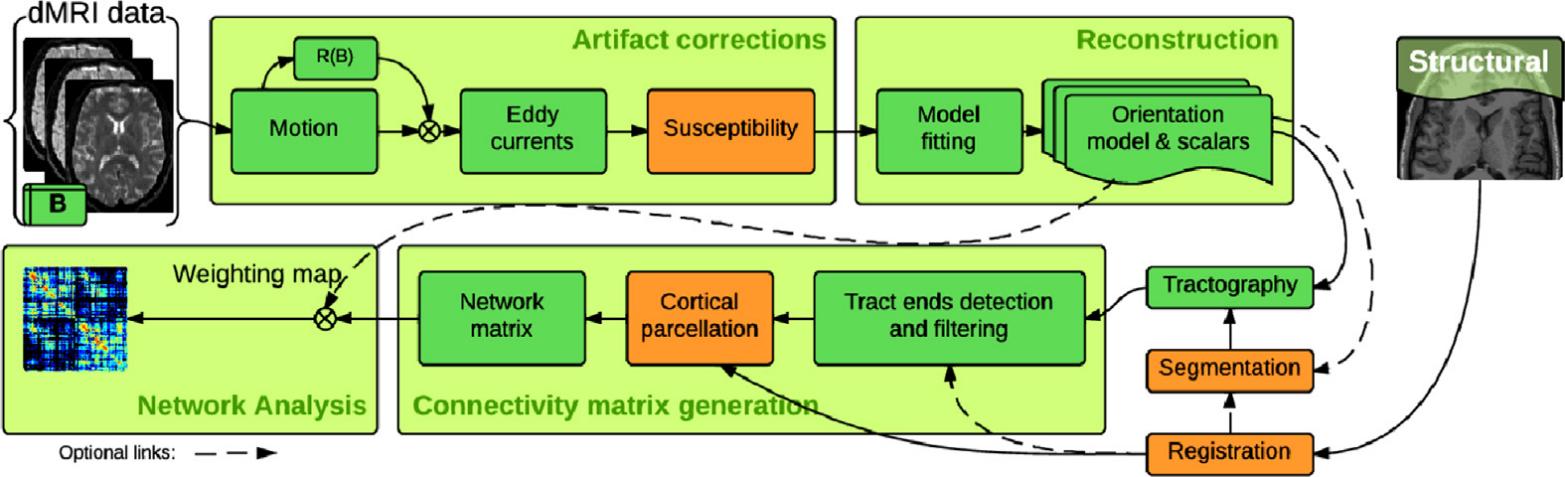
The data for the verification and validation of the elements involved in the connectome extraction are valuable due to the absence of reference-standards. The analysis of structural connectivity networks extracted from dMRI data involves a convoluted processing flow comprising a large set of chained computational tools. Unit-test verification and validation of these tasks is crucial to assess the reliability of the whole process, and a challenging effort due to the lack of gold standards. In [1] a joint registration and segmentation method that implicitly tackles with the susceptibility-derived distortion artifact is proposed, and evaluated on the surfaces as a surrogate of the goodness of the cortical parcellation. The involved elements in [1] are denoted with orange-color boxes. In this paper, we provide the data and the software instruments used to generate a “*golden*”-standard required in the evaluation of the segmentation and registration task.

Here we share phantom data for MRI registration and segmentation validation, the software instrumentation, and the figures and tables generated by the reporting utilities of our evaluation framework. We also extend the mathematical formulations of a simultaneous segmentation and registration tool called *regseg* [1] designed to be included in processing workflows like the one presented in Fig. 1.

## 2 Experimental design, materials and methods

**Fig. 2:**
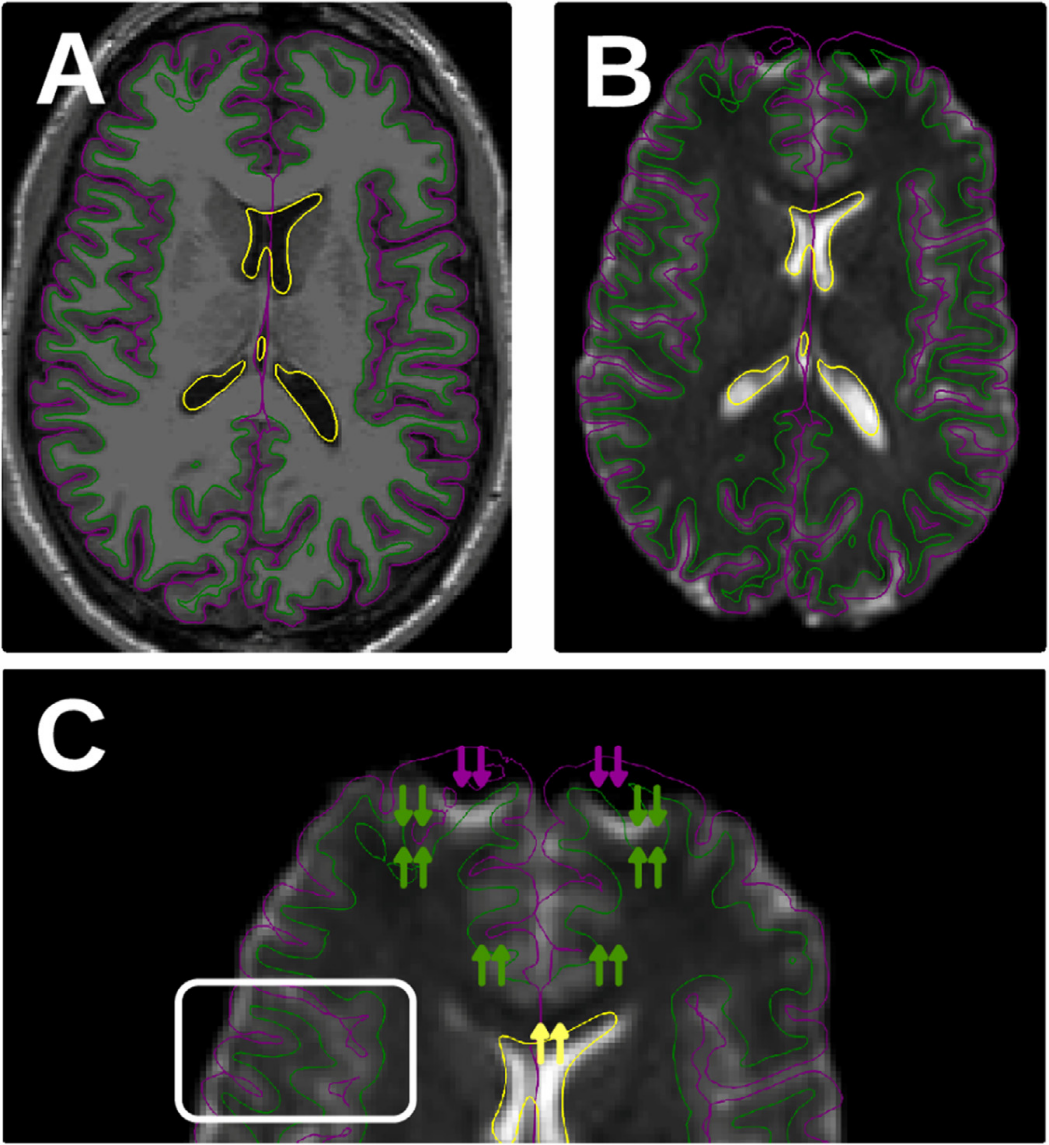
Susceptibility distortions are challenging in dMRI. The artifact causes a misalignment of the structures of the brain (represented by contours overlaid on the T1-weighted -T1w- image of panel A) and the dMRI data (as depicted in panel B). In panel C we present a close-up of the frontal lobe of the diffusion image, where the warping of the echo-planar image (EPI) produces a mismatch with respect the “anatomically-correct” surfaces extracted from the T1w image. The warping is aligned with the phase-encoding (PE) direction of the image. In this case (panels B, C) the PE direction is the anterior-posterior axis. Since the distortion is related to the inhomogeneity of the field inside the scanner, some regions are not excessively affected by the artifact (white box in panel C). In this data paper, the methodology and instruments to generate “*a priori*” known distortions from real subjects that can be used as “*golden*”-standard in the validation of registration and segmentation processing tools for diffusion MRI.

**Fig. 3:**
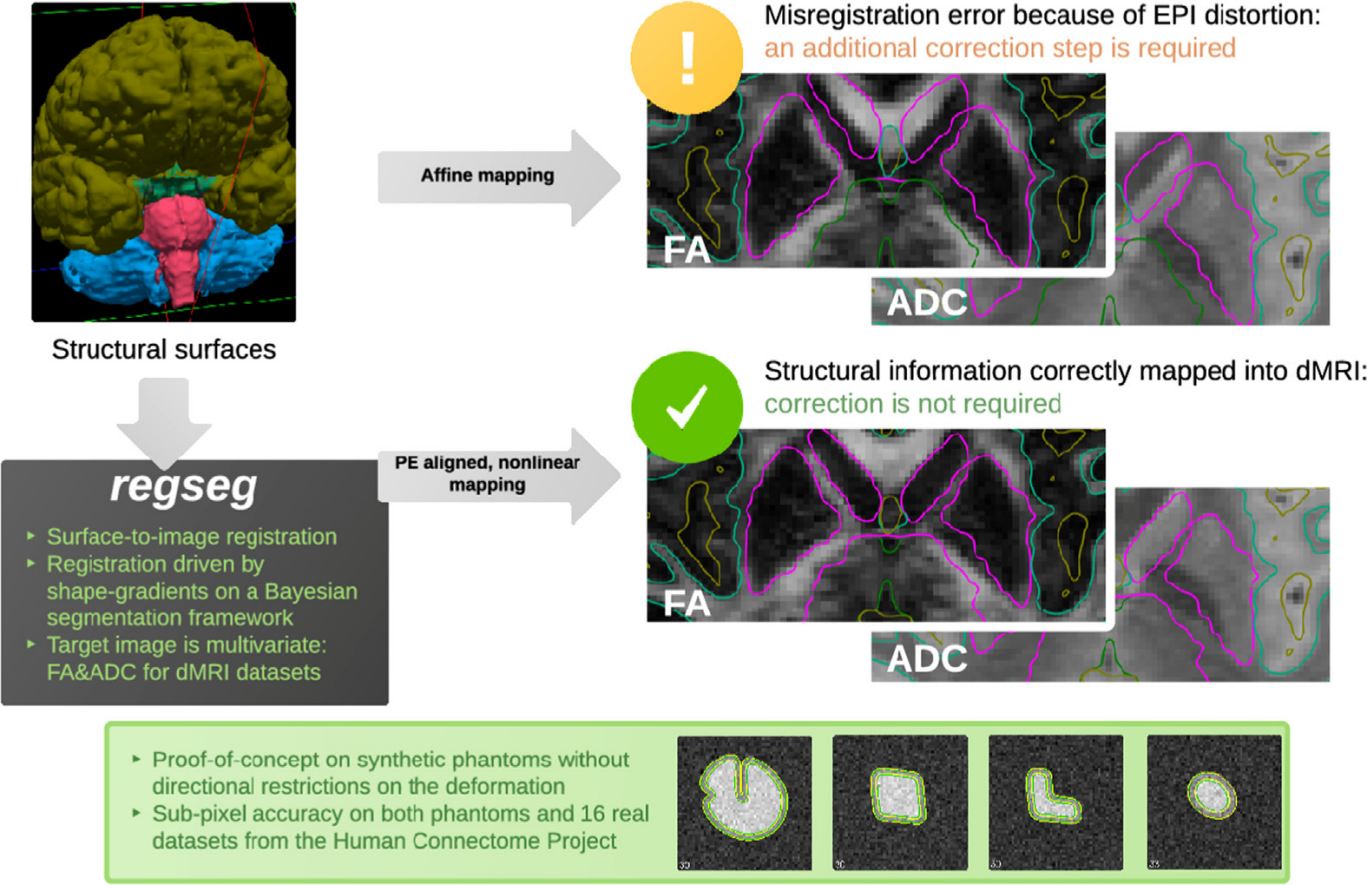
Experimental design and the regseg tool. The proposed tool performs simultaneous segmentation and registration of dMRI features (the FA and the ADC maps) through a nonlinear mapping aligned with the phase-encoding (PE) axis of the echo-planar images (EPI). This data paper provides detailed information with figures, graphs and text of how the necessary “*golden*”-standard to validate regseg was obtained, and the mathematical foundations of the method.

In order to assess the performance of segmentation and registration methods, we propose in [1] the following general evaluation protocol: 1) Extract the set of reference surfaces, as in Fig. 2A; 2) Compute a realistic field of displacements which is applied to generate warped images like those presented in Fig. 2B for the evaluation purposes; 3) Execute the task under test; and 4) Perform a visual assessment and compute the error metrics. This generic experimental design is illustrated in Fig. 3 for the particular application presented in [1].

In the supplementary document, the method presented in [1] is described in deeper detail in Section S1. Then, in Section S2 the specific details on the practical use of the tool are provided, including the description of the different parameters and options available, and the reporting panels generated by the tool to ensure the correct performance, like the one presented in Fig. S2. Section S3 describes the processing workflows and sub-workflows that are the building blocks of the overall experimental design. Since *regseg* proposes a segmentation model appropriate for the FA and ADC maps derived from dMRI images, this model is described in Section S4, including the plots evidencing the evolution of the model through the registration-segmentation process. Finally, Section S5 provides a mosaic visualization of the results of the registration process performed on the sample of subjects for evaluation used in [1], including the comparison to the alternate method for registration.

We provide four digital phantoms for the validation of registration and segmentation methods. These phantoms show different shapes, some are designed to be challenging for segmentation methods and others are challenging in registration. Software instruments provided within the Github repository are written in Python, using the nipype framework [3] to ensure their reproducibility and maintenance.

Reporting elements include graphs and figures generated automatically with matplotlib [4], and in-house modifications^2^ of *seaborn* [5].

## Footnotes

1 https://github.com/oesteban/RegSeg.

2 https://github.com/oesteban/seaborn.
