# Supplementary material for: Data on the verification and validation of segmentation and registration methods for diffusion MRI
Source: Data Brief. 2016 Jul 2;8:871–6. doi: 10.1016/j.dib.2016.06.049 (PMC4957576; doi:10.1016/j.dib.2016.06.049)
Supplement: Supplementary file 1 — Supplementary material [file mmc1.doc]

The authors declare that there is no conflict of interest
