# Supplementary material for: Data on the verification and validation of segmentation and registration methods for diffusion MRI
Source: Data Brief. 2016 Jul 2;8:871–6. doi: 10.1016/j.dib.2016.06.049 (PMC4957576; doi:10.1016/j.dib.2016.06.049)
Supplement: Supplementary file 2 — Supplementary material [file mmc2.pdf]

# Supplemental Materials:
# *Surface-driven registration method for the structure-informed segmentation of diffusion MR images*

Esteban O., Zosso D., Daducci A., Bach-Cuadra M.,
Ledesma-Carbayo MJ., Thiran JP, and Santos A.

10.1016/j.neuroimage.2016.05.011

# S1 Extensions to the mathematical formulation of the methods

## S1.1 Computing the gradients of shape-priors

The computation of gradients at the locations of the active contours in the instant $t$ is based on the work of Herbulot et al. (2006). Let $F(\mathbf{r})$ be an "arbitrary" function over the image domain $\Omega = \Omega_l \cup \Omega_m$ split in two regions $l$ and $m$, and $\Gamma_{l,m}$ a closed boundary between them. We now derive the domain integral w.r.t. $t$:

$$\frac{\partial}{\partial t}\int_{\Omega} F(\mathbf{r})d\mathbf{r} = \int_{\Omega}\frac{\partial}{\partial t}F(\mathbf{r})d\mathbf{r} - \int_{\Gamma_{l,m}} F(\mathbf{r})\left\langle \frac{\partial \Gamma_{l,m}}{\partial t}, N_{\Gamma_{l,m}}\right\rangle d\mathbf{r}, \quad \text{(SM1)}$$

where $\left\langle \frac{\partial \Gamma_{l,m}}{\partial t}, N_{\Gamma_{l,m}}\right\rangle$ is the projection of the boundary movement on the unit inward normal $N_{\Gamma_{l,m}}$. Assuming that the region descriptors $\{\boldsymbol{\mu}_l, \boldsymbol{\Sigma}_l\}$ vary slowly enough, we can consider that $\frac{\partial}{\partial t}F(\mathbf{r}) = 0$ and thus:

$$\frac{\partial}{\partial t}\int_{\Omega} F(\mathbf{r})d\mathbf{r} = -\int_{\Gamma_{l,m}} F(\mathbf{r})\left\langle \frac{\partial \Gamma_{l,m}}{\partial t}, N_{\Gamma_{l,m}}\right\rangle d\mathbf{r}. \quad \text{(SM2)}$$

The equation (SM2) is discretized as follows. First, the surface between limiting regions $\{l, m\}$, $\Gamma_{l,m}$ is explicitly represented by a discrete set of vertices $\mathbf{v}_i$, with $i \in \{0, \dots, N_p - 1\}$. Consequently, the inwards normal of the surface $N_{\Gamma_{l,m}}$ is represented by the discrete set of normals $\hat{\mathbf{n}}_i$ at each vertex of the mesh. The resulting summation is, therefore, discrete and the integral operator is replaced by the sum:

$$\begin{aligned}\frac{\partial}{\partial t}\int_{\Omega} F(\mathbf{r})d\mathbf{r} &= \underbrace{\cancel{\int_{\Omega}\frac{\partial}{\partial t}F(\mathbf{r})d\mathbf{r}}}_{\text{Functional's evolution}} - \underbrace{\int_{\Gamma_{l,m}} F(\mathbf{r})\left\langle \frac{\partial \Gamma_{l,m}}{\partial t}, N_{\Gamma_{l,m}}\right\rangle d\mathbf{r}}_{\text{Shape's evolution}} \\ &= -\sum_p \frac{1}{A_p}\sum_i a_i\, F(\mathbf{v}_i)\left\langle \underbrace{\frac{\partial \mathbf{v}_i}{\partial t}}_{\text{speed of } \mathbf{v}_i}, \hat{\mathbf{n}}_i\right\rangle. \end{aligned} \quad \text{(SM3)}$$

where $a_i$ is the area corresponding to vertex $\mathbf{v}_i$, and $A_p = \sum_i a_i$ is the total area of surface $p$. In the following, we will refer as $w_{p,i} = a_i/A_p$ to the area contribution of $\mathbf{v}_i$ to the total area of the surface it belongs to. For simplicity, the sum over $p$ can be also removed, as the vertices belong to only one of the total $P$ contours

Then, the speed of $\mathbf{v}_i$ is discretized using the artificial time-step parameter $\delta$, as the displacement $\frac{\partial \mathbf{v}_i}{\partial t} = \mathbf{v}_i(\delta = t+1) - \mathbf{v}_i(\delta = t)$:

$$\frac{\partial}{\partial t}\int_{\Omega} F(\mathbf{r})\, d\mathbf{r} = -\sum_i w_{p,i} F(\mathbf{v}_i)\frac{\partial \mathbf{v}_i}{\partial t}\cdot \hat{\mathbf{n}}_i. \quad \text{(SM4)}$$

Since the energy functional is defined over competing regions, the displacement of $\mathbf{v}_i$ will cause an energy exchange between the limiting regions, and therefore $F(\mathbf{r})$ must be split in two terms, $F_{in}(\mathbf{r})$ corresponding to the interior region and $F_{out}(\mathbf{r})$ to the exterior:

$$\frac{\partial}{\partial t}\int_{\Omega} F(\mathbf{r})\, d\mathbf{r} = -\sum_i \frac{\partial \mathbf{v}_i}{\partial t}\cdot \underbrace{w_{p,i}\left[F_{out}(\mathbf{v}_i) - F_{in}(\mathbf{v}_i)\right]\hat{\mathbf{n}}_i}_{\bar{s}_i \text{ in Figure 1}}. \quad \text{(SM5)}$$

## S1.2 Gradient-descent optimization

The energy functional to be optimized in *regseg* is presented in Eq. 7. After the simplifications described in equation (A.1) of Appendix 1, we obtain the following energy functional:

$$E(\mathbf{u}) = C + \underbrace{\sum_l \int_{\Omega_l} \mathcal{D}_l^2(\mathbf{f}')\, d\mathbf{r}}_{\text{Data term } (E_{data})} + \underbrace{\int_{\Omega} \left[\boldsymbol{\alpha}\cdot \mathbf{u}^{\circ 2} + \boldsymbol{\beta}\cdot (\nabla \mathbf{u})^{\circ 2}\right]\, d\mathbf{r}}_{\text{Regularization term } (E_{reg})}. \quad \text{(SM6)}$$

To search for the minimum of $E(\mathbf{u})$ w.r.t. the coefficients $\mathbf{u}_k$, we use a gradient descent strategy. In Eq. (9) we introduced the derivative of (SM6):

$$\frac{\partial E(\mathbf{u})}{\partial \mathbf{u}_k} = \frac{\partial}{\partial \mathbf{u}_k}\Big\{\sum_l \int_{\Omega_l} \mathcal{D}_l^2(\mathbf{f}')\, d\mathbf{r} + \int_{\Omega}\frac{1}{2}[\boldsymbol{\alpha}\cdot \mathbf{u}^{\circ 2} + \boldsymbol{\beta}\cdot (\nabla \mathbf{u})^{\circ 2}]\, d\mathbf{r}\Big\}. \quad \text{(SM7)}$$

We split (SM7) in the derivatives of its data and regularization terms. Let $\frac{\partial E_{data}}{\partial \mathbf{u}_k} = \mathbf{g}_k$ for simplicity, we compute the derivative of the data term and discretize the domain $\Omega$ as follows:

$$\mathbf{g}_k = \frac{\partial}{\partial \mathbf{u}_k}\Big\{\sum_l \int_{\Omega_l} \mathcal{D}_l^2(\mathbf{f}')\Big\} = \frac{\partial}{\partial \mathbf{u}_k}\Big\{\sum_l \sum_{\mathbf{x}\in\Omega_l} \mathcal{D}_l^2(\mathbf{f}')\Big\}, \tag{SM8}$$

where we can apply the shape-gradients (SM5) introduced in subsection S1.1, and ultimately avoid implementing level sets:

$$\mathbf{g}_k = \sum_i \left\langle \frac{\partial \mathbf{v}_i'}{\partial \mathbf{u}_k}, \bar{s}_i' \right\rangle, \tag{SM9}$$

$$\text{with } \bar{s}_i' = -w_i \left[\mathcal{D}_{out}^2(\mathbf{f}_\mathbf{i}') - \mathcal{D}_{in}^2(\mathbf{f}_\mathbf{i}')\right] \hat{\mathbf{n}}_i, \tag{SM10}$$

$$\text{and } \frac{\partial \mathbf{v}_i'}{\partial \mathbf{u}_k} = \frac{\partial}{\partial \mathbf{u}_k}\left\{\mathbf{v}_i + \sum_k \psi_k(\mathbf{v}_i)\mathbf{u}_k\right\} = \psi_k(\mathbf{v}_i)\,\hat{\mathbf{e}}. \tag{SM11}$$

where $\hat{\mathbf{e}}$ is the coordinates system's unit vector. Therefore, the shape gradients projected to the grid of B-spline control points is:

$$\mathbf{g}_k = -\sum_i \bar{s}_i \cdot \psi_k(\mathbf{v}_i)\,\hat{\mathbf{e}} = -\sum_i \mathbf{g}_{i,k}. \tag{SM12}$$

It is also necessary to obtain and discretize the derivatives of the regularization term of (SM7):

$$\frac{\partial E_{reg}(\mathbf{u})}{\partial \mathbf{u}_k} = \frac{\partial}{\partial \mathbf{u}_k}\left\{\int_\Omega \frac{1}{2}[\boldsymbol{\alpha}\cdot\mathbf{u}^{\circ 2} + \boldsymbol{\beta}\cdot(\nabla\mathbf{u})^{\circ 2}]\,d\mathbf{r}\right\} = \boldsymbol{\alpha}\cdot\mathbf{u}_k - \boldsymbol{\beta}\cdot\Delta\mathbf{u}_k. \tag{SM13}$$

Inserting (SM12) and (SM13) into (SM7) we get the final evolution equation:

$$\frac{\partial \mathbf{u}}{\partial t} \propto -\frac{\partial E(\mathbf{u})}{\partial \mathbf{u}_k},$$
$$\frac{\partial \mathbf{u}}{\partial t} \propto -\Big(\mathbf{g}_k - \boldsymbol{\alpha}\cdot\mathbf{u}_k + \boldsymbol{\beta}\cdot\Delta\mathbf{u}_k\Big). \tag{SM14}$$

## S1.3 Obtaining the update equation

To solve the differential equation in (SM14), we use a semi-implicit Euler scheme, referring to the discrete step size as $\delta$ and where the shape-gradients $\mathbf{g}_k$ are explicit:

$$\mathbf{u}_k^{t+1} = \mathbf{u}_k^t + \delta\Big(-\mathbf{g}_k^t - (\boldsymbol{\alpha} - \boldsymbol{\beta}\Delta)\,\mathbf{u}_k^{t+1}\Big)$$
$$(1 + \delta\,\boldsymbol{\alpha} - \delta\,\boldsymbol{\beta}\Delta)\,\mathbf{u}_k^{t+1} = \mathbf{u}_k^t - \delta\,\mathbf{g}_k^t \tag{SM15}$$

This expression is easily translated into Fourier domain as follows:

$$\mathbf{u}_k^{t+1} = \mathcal{F}^{-1}\left\{\frac{\mathcal{F}\{\delta^{-1}\,\mathbf{u}_k^t - \mathbf{g}_k^t\}}{\mathcal{F}\{(\delta^{-1} + \boldsymbol{\alpha})\,I - \boldsymbol{\beta}\Delta\}}\right\}, \tag{SM16}$$

where $I$ denotes the identity operator. Here, we rewrite the Laplacian as a linear combination of the identity and shift operators:

$$\Delta = \left\{\sum_{d=1}^n \mathcal{S}_d^- + \mathcal{S}_d^+\right\} - 2n\mathcal{I} \tag{SM17}$$

where $\mathcal{S}_d^\pm$ stands for the forward (+) and backward (−) shift operator along axis $d$, of which the Fourier transform is found easily as

$$\mathcal{F}\{\mathcal{S}_d^\pm\} = e^{\pm i\omega_d}, \tag{SM18}$$

where $\omega_d$ is the normalized pulsation along direction $d$. Accordingly, the Fourier transform of the discrete Laplacian is found as

$$\mathcal{F}\{\Delta\} = \sum_d e^{-i\omega_d} + e^{i\omega_d} - 2n = n\left(\sum_d \cos(\omega_d) - 2\right) \tag{SM19}$$

The remaining transforms are trivial or can be computed using FFT as in (Estellers et al., 2012).

# S2 Parameter settings and implementation details of *regseg*

## S2.1 Implementation

## S2.2 General

The *regseg* registration and segmentation tool is written in C++, using ITK-4.6 (Ibanez et al., 2005) as implementation core. We designed a modular implementation, enabling multithreading in several pieces of the software, as the process is computationally expensive. The tool generates a log-file in JSON format to easily inter-operate with secondary tools (such as the convergence report generation, subsection S2.6). The evaluation workflow is independent, and implemented using *nipype* (Gorgolewski et al., 2011).

## S2.3 Efficient interpolation using sparse matrices

During the registration process, every iteration requires computing the product of all the gradients $\mathbf{g}_k$ associated to the control point $k$, and computed at the current position $\mathbf{v}_i$ by the corresponding weights $\psi_{ik} = \psi_k(\mathbf{v}_i)$ of interpolating functions (SM12). In order to optimize multiplications and summations, all the $\psi_{ik}$ are collected in a matrix $\mathbf{\Psi} = (\psi_{ik})$. Given the limited support of the basis function $\psi$, $\mathbf{\Psi}$ will hold the property of being sparse, as only few $\psi_{ik} > 0$ in the surroundings of $\mathbf{v}_i$. Then, the gradients $\mathbf{g}_k$ are easily computed using the matrix product:

$$\left(\mathbf{g}_k\right) = \mathbf{\Psi} \cdot \left(w_i\, \bar{s}_i\right)^T \tag{SM20}$$

As these weights can be computed once in the beginning of the process and they do not change along it, $\mathbf{\Psi}$ can be pre-cached.

## S2.4 Assessment of the segmentation model

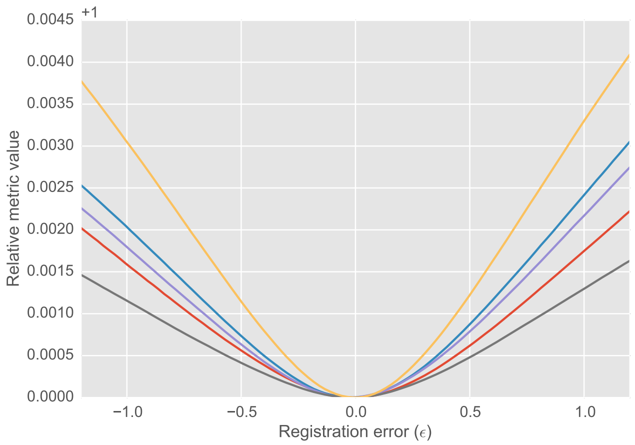


Figure S1: **Assessment of the segmentation model**: Preliminarily, we investigated the aptness of the segmentation model. For five test datasets, we uniformly sampled the space of distortions $\hat{U} = \epsilon \cdot U_{true} = \mathbf{r} + \epsilon\, u_{\text{PE}}$ (with $\epsilon \in [-1.1, 1.1]$ and $u_{\text{PE}}$ from Equation 13 in the paper), and evaluated the data-term of the cost function (SM6). The metric consistently displayed its minimum at the ground-truth location ($\epsilon = 0.0$) for all the cases, indicating that the segmentation model is appropriate for registration.

## S2.5 Interface and Settings

**Command-line interface** The command line interface of *regseg* supports general settings and level-wise settings. For each multi-resolution level, its corresponding settings are added between brackets.

```
regseg -F fa.gz adc.nii.gz -P white.vtk pial.vtk -o myprefix [ -a 0.00000 -b 0.00000 --convergence-energy -t
     1.0e-06 -w 60 --adaptative-descriptors --grid-spacing 16.0 -i 500 -s 0.001] [ -a 0. -b 0. --convergence
    -energy -t 1.e-08 -w 5 --grid-spacing 8.0 -i 250 -s 0.01]
```

It is possible to get the description of available options running `regseg -h` :

```
Usage:

General options:
  -h [ --help ]                       show help message
  -F [ --fixed-images ] arg           fixed image file
  -P [ --surface-priors ] arg         shape priors
  -T [ --surface-target ] arg         final shapes to evaluate metric (only
                                      testing purposes)
  -M [ --fixed-mask ] arg             fixed image mask
  -L [ --transform-levels ] arg       number of multi-resolution levels for
```

```
                                       the transform
  -o [ --output-prefix ] arg (=regseg)  prefix for output files
  -l [ --logfile ] arg                  log filename
  -v [ --monitoring-verbosity ] arg (=1)
                                        verbosity level of intermediate results
                                        monitoring ( 0 = no output; 5 = verbose
                                        )

Optimizer options (by levels):
  -a [ --alpha ] arg              alpha value in regularization
  -b [ --beta ] arg               beta value in regularization
  -s [ --step-size ] arg          step-size value in optimization
  -g [ --gradient-scales ] arg    alpha value in regularization
  -r [ --learning-rate ] arg      learning rate to update step size
  -i [ --iterations ] arg         number of iterations
  -w [ --convergence-window ] arg number of iterations of convergence window
  -t [ --convergence-thresh ] arg convergence value
  --grid-size arg                 size of control points grid
  --grid-spacing arg              spacing between control points
  -u [ --update-descriptors ] arg frequency (iterations) to update descriptors
                                  of regions (0=no update)
  --adaptative-descriptors        recomputes descriptors more often at the
                                  beginning of the process
  --convergence-energy            disables lazy convergence tracking: instead
                                  of fast computation of the mean norm of the
                                  displacement field, it computes the full
                                  energy functional

Functional options (by levels):
  --smoothing arg               apply isotropic smoothing filter on target
                                image, with kernel sigma=S mm.
  --smooth-auto                 apply isotropic smoothing filter on target
                                image, with automatic computation of kernel
                                sigma.
  --uniform-bg-membership       consider last ROI as background and do not
                                compute descriptors.
  -d [ --decile-threshold ] arg set (decile) threshold to consider a computed
                                gradient as outlier (ranges 0.0-0.5)
```

***Nipype* interface** Our registration algorithm is released with a *nipype Interface* packaged in `pyacwereg.interfaces.acwereg`. This interface has been comprehensively used in the evaluation workflows.

```python
from pyacwereg.interfaces.acwereg import ACWEReg
regseg = ACWEReg()
regseg.inputs.in_fixed = ['T1w.nii.gz', 'T2w.nii.gz']
regseg.inputs.in_pior = ['csf.vtk', 'white_lh.vtk', 'white_rh.vtk',
                         'pial_lh.vtk', 'pial_rh.vtk']
ifresult = regseg.run()
```

## S2.6 Convergence evidencing

In order to track the evolution of the registration process, several internal variables are saved in the JSON log-file. Using the JSON log-file as input for the *nipype Interface* `ACWEReport`, it is straightforward to obtain a visual assessment document presenting the convergence.

```python
from pyacwereg.interfaces.acwereg import ACWEReport
report = ACWEReport()
report.inputs.in_log = 'myprefix.log'
ifresult = report.run()
```

Online checking is also possible as the algorithm writes to the standard output as well. A sample report is found in Figure S2.

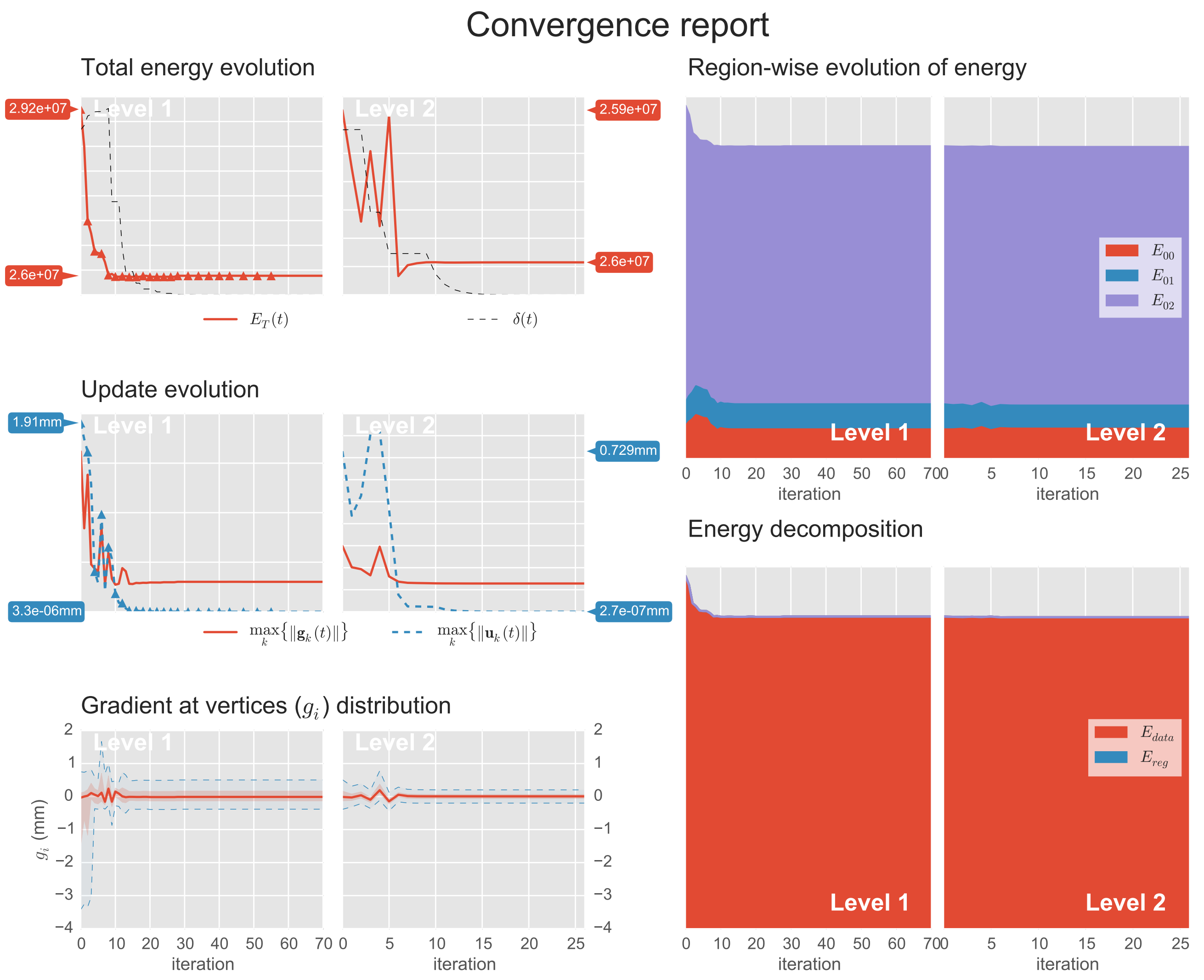


Figure S2: The evolution of the registration and segmentation process can be checked using the *Convergence report*, easily generated using the appropriate *nipype Interface*. The report comprehends several plots tracking the evolution of the algorithm and several features to help researchers tune up the algorithm in their application.

# S3 Instruments for evaluation

This work is supported by two *nipype Workflows* in order to ensure the reproducibility of the results. All the intermediate results and figures in this paper have been encapsulated into the workflows and are available in (Esteban and Zosso, 2015).

An overview of the workflow applied in phantoms is presented in the Figure 2 of the paper. Subsequently, Figure 3 reproduces the evaluation on real data. The two figures have been extremely simplified for the best of visualization. In this section, we review the main elements of the evaluation pipelines.

## S3.1 Extraction of structural information

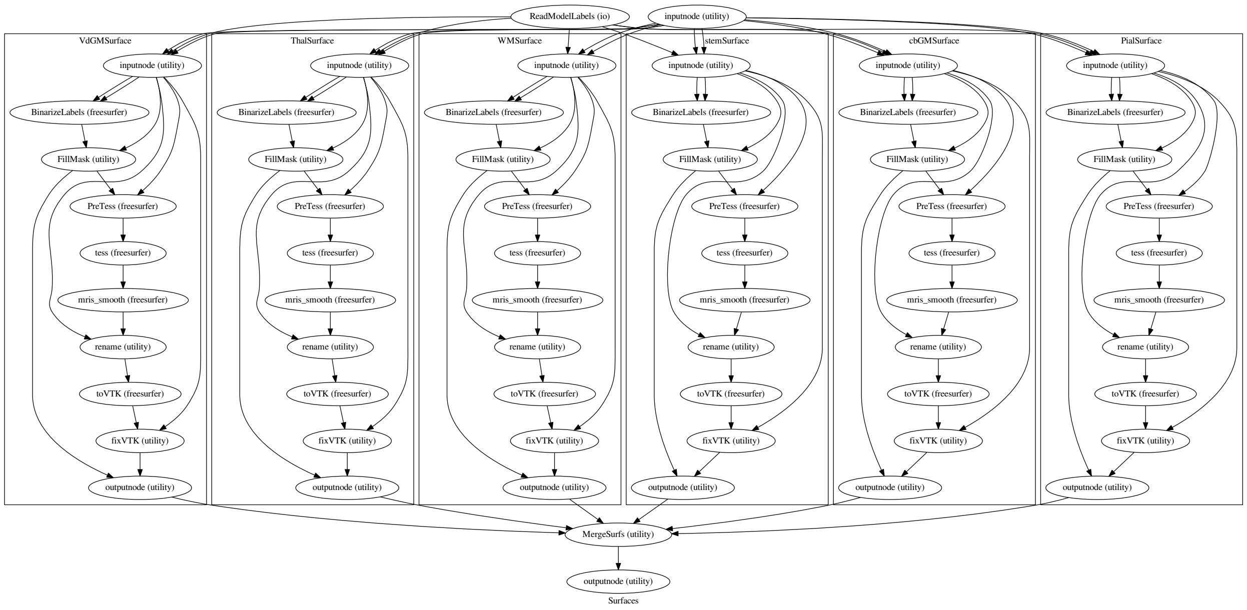


Figure S3: **Generating Surfaces**. Each surface extracted in the structural reference (in both phantoms and real datasets) is obtained with a unitary pipeline, for instance, the *PialSurface* block. In this figure, the composite workflow to extract the six surfaces that define the segmentation model (Figure S7) is represented.

## S3.2 Processing diffusion MRI data

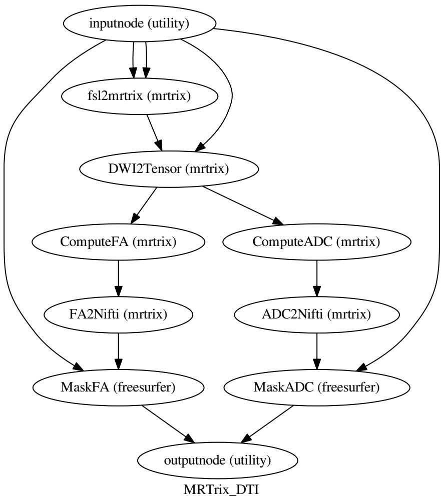


Figure S4: **Workflow to generate FA and ADC maps from dMRI data**. We use *MRtrix* (Tournier et al., 2012) to generate a DTI from data and computing the two feature maps of interest (FA and ADC). This workflow is performed in both undistorted and warped data.

## S3.3 T2-weighted registration to *b0* -based (T2B) method

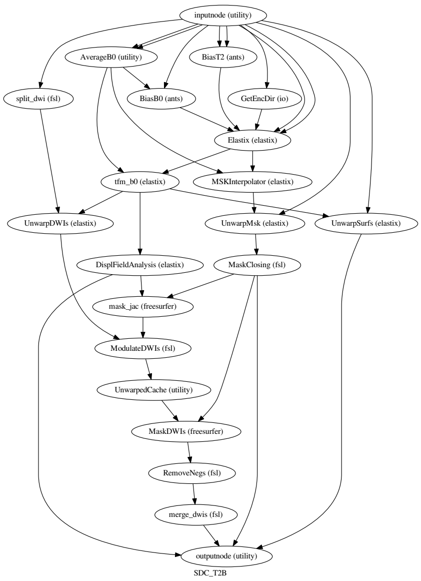


Figure S5: **In-house implementation of the T2B method**. Each surface extracted in the structural reference (in both phantoms and real datasets) is obtained with a unitary pipeline, for instance, the *PialSurface* block. In this figure, the composite workflow to extract the six surfaces that define the segmentation model (Figure S7) is represented.

## S3.4 Complete workflow for evaluation on real data

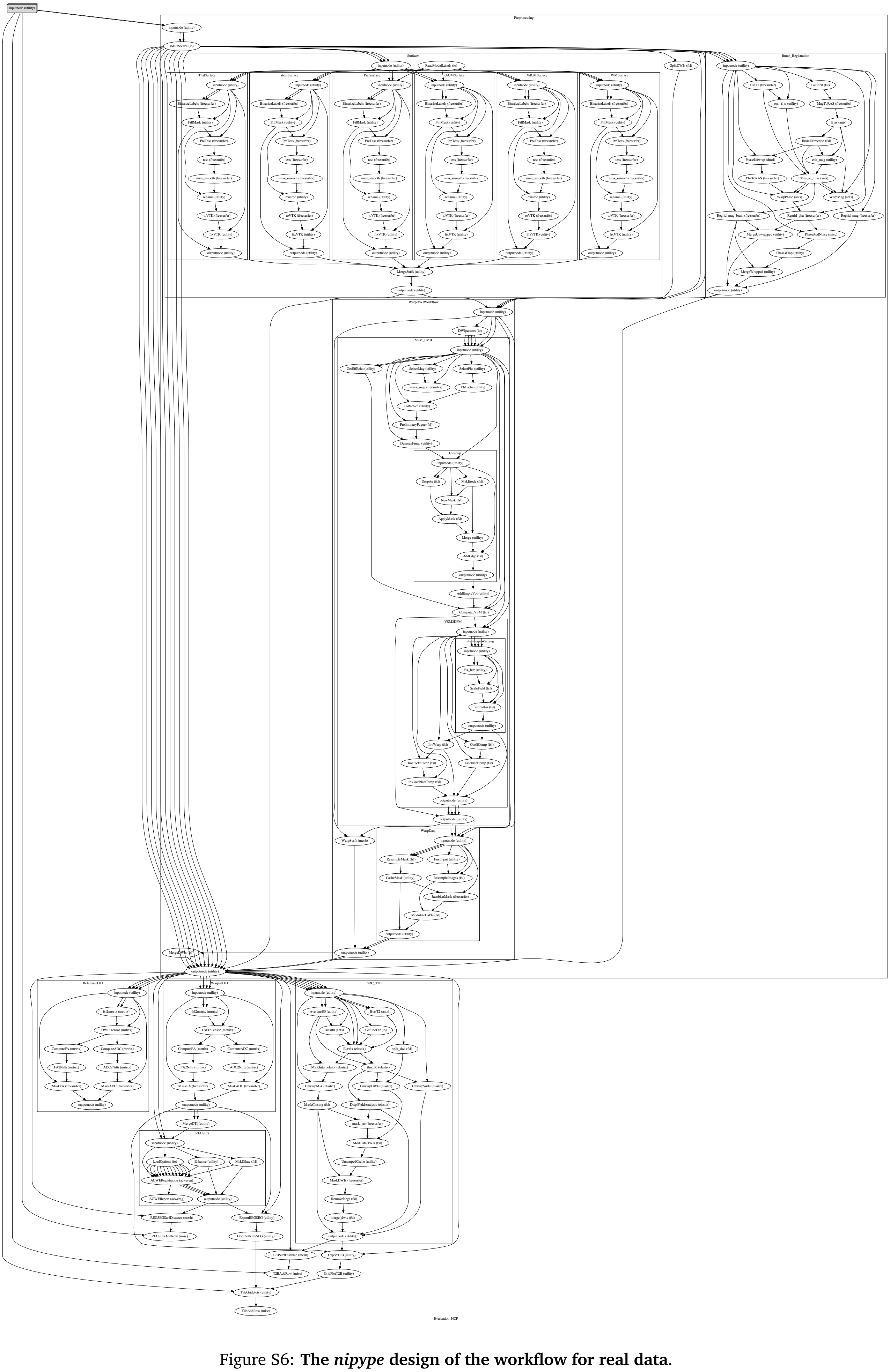


Figure S6: **The *nipype* design of the workflow for real data.**

# S4 Model considerations

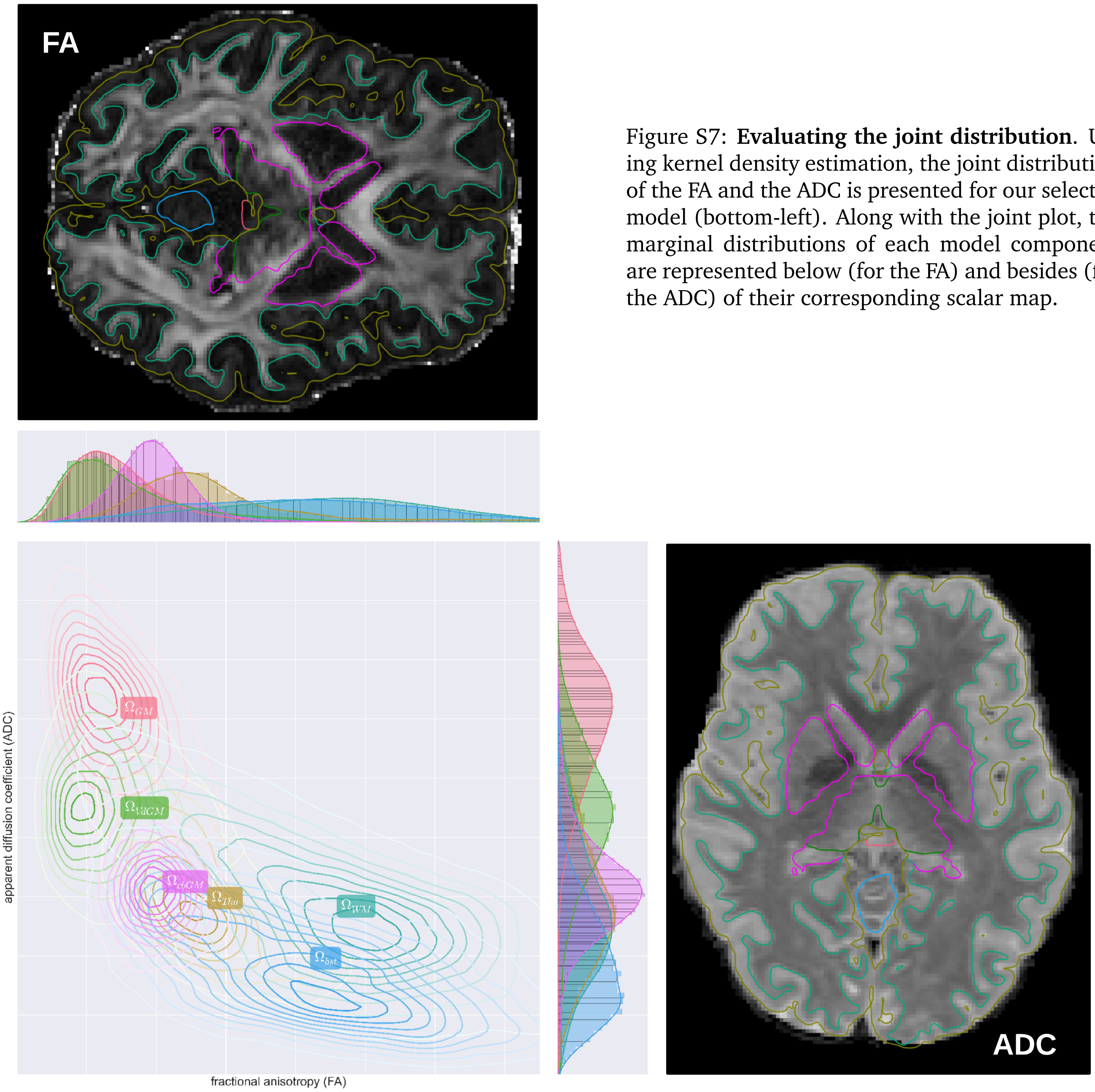


Figure S7: **Evaluating the joint distribution**. Using kernel density estimation, the joint distribution of the FA and the ADC is presented for our selected model (bottom-left). Along with the joint plot, the marginal distributions of each model component are represented below (for the FA) and besides (for the ADC) of their corresponding scalar map.

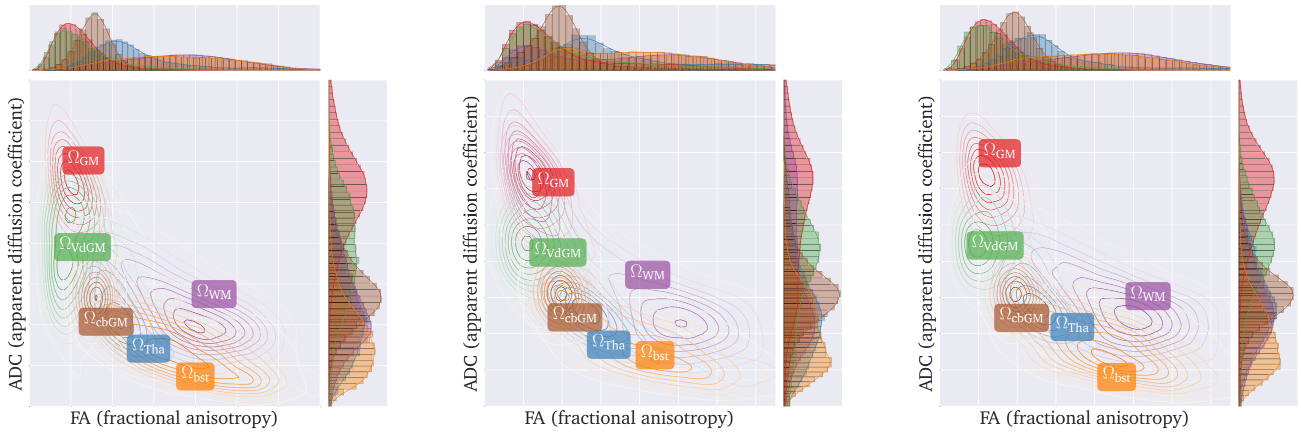


Figure S8: Detail of the evolution of the model (all tissue types).

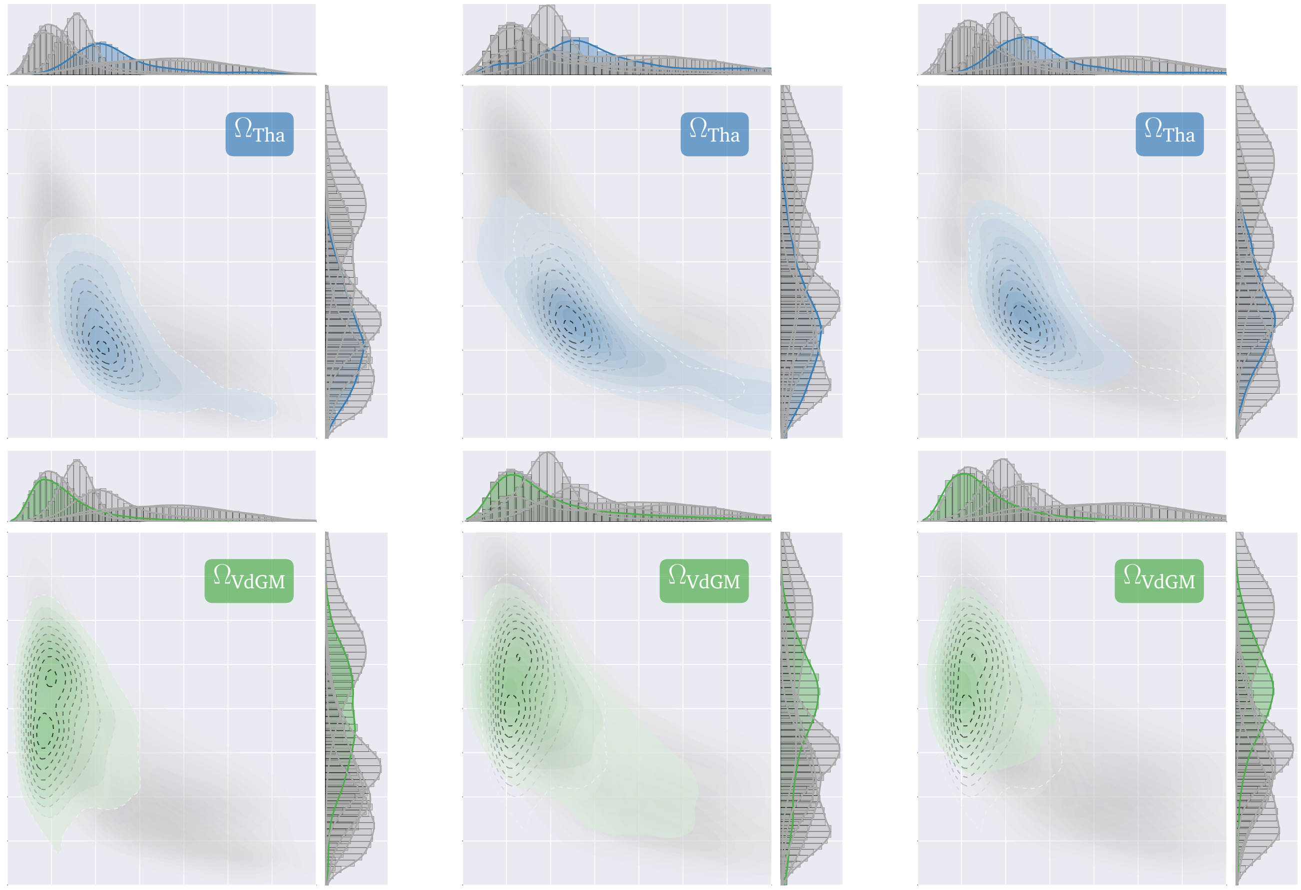

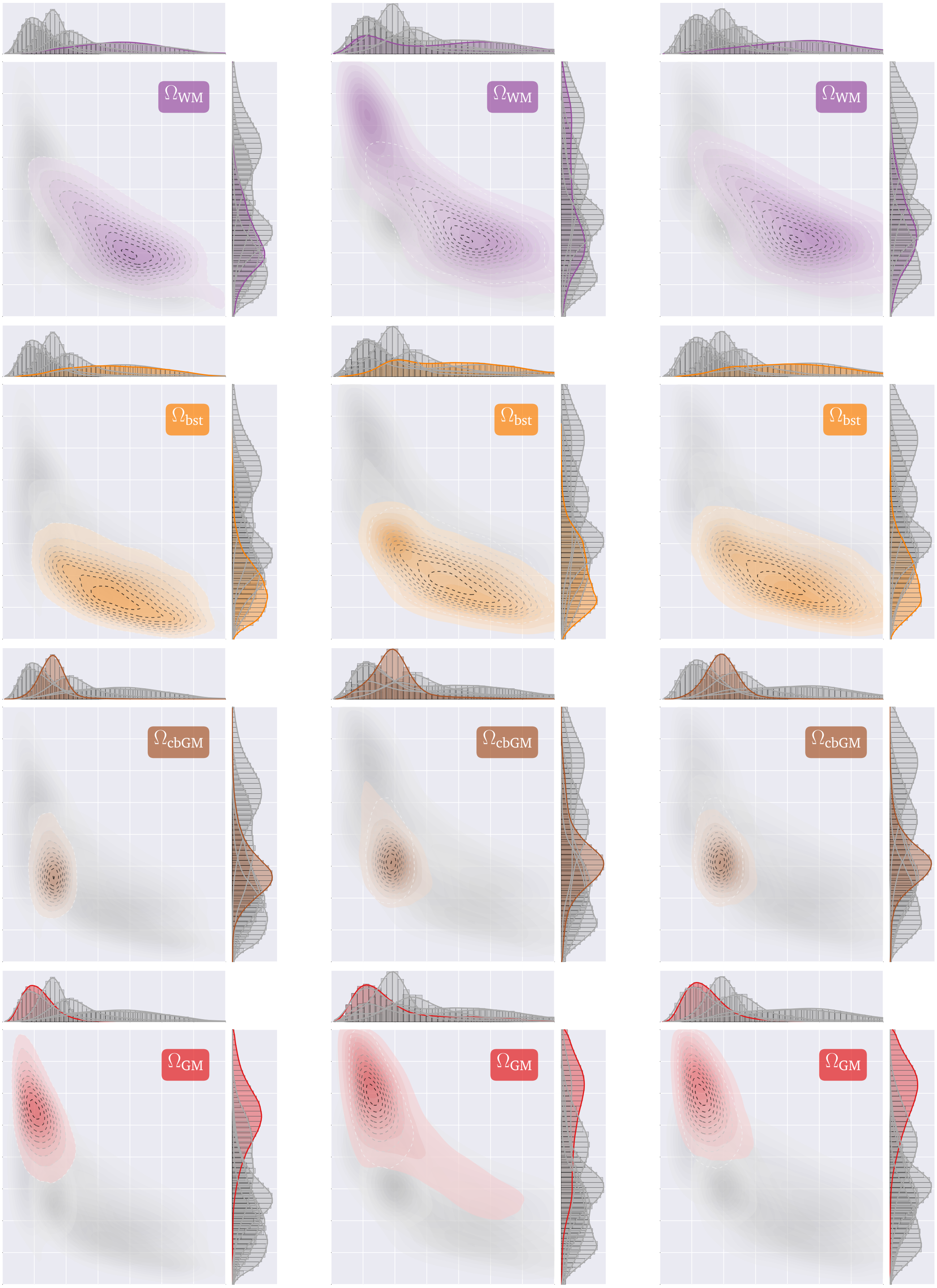


Figure S9: Detail of the evolution of the model (tissue-wise distribution).

# S5 Extended results on real data

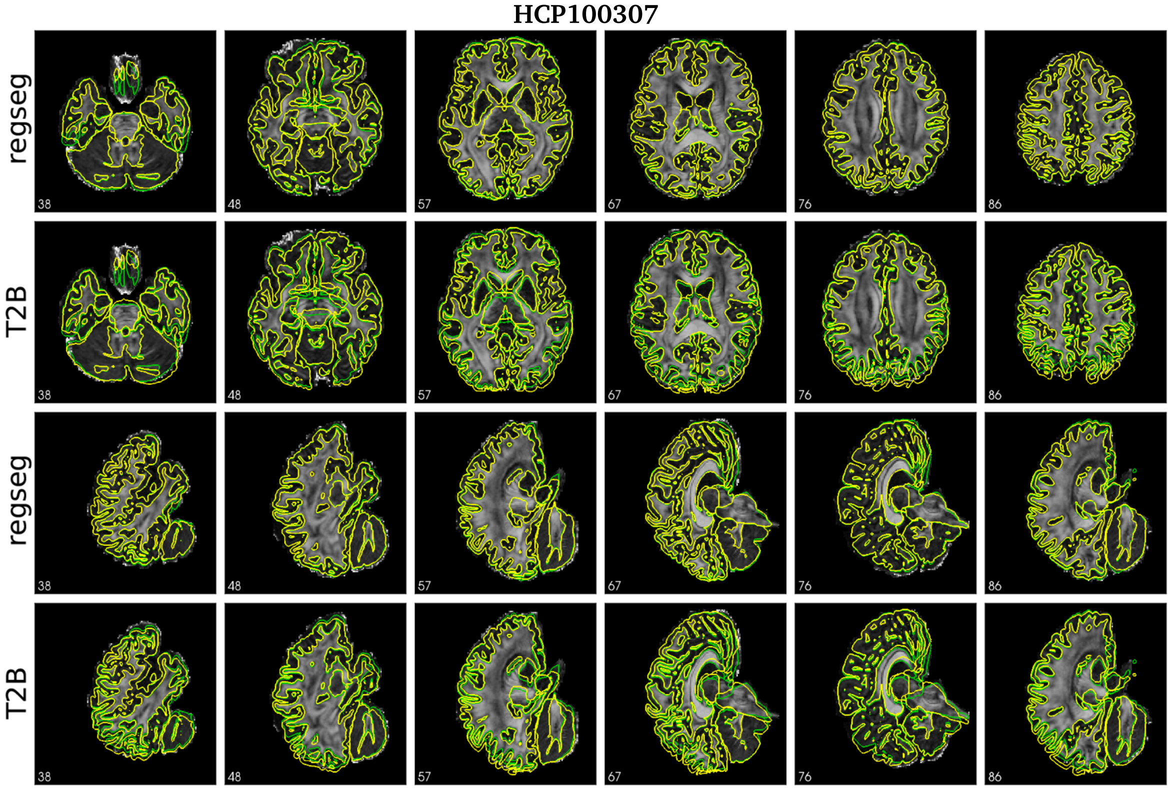


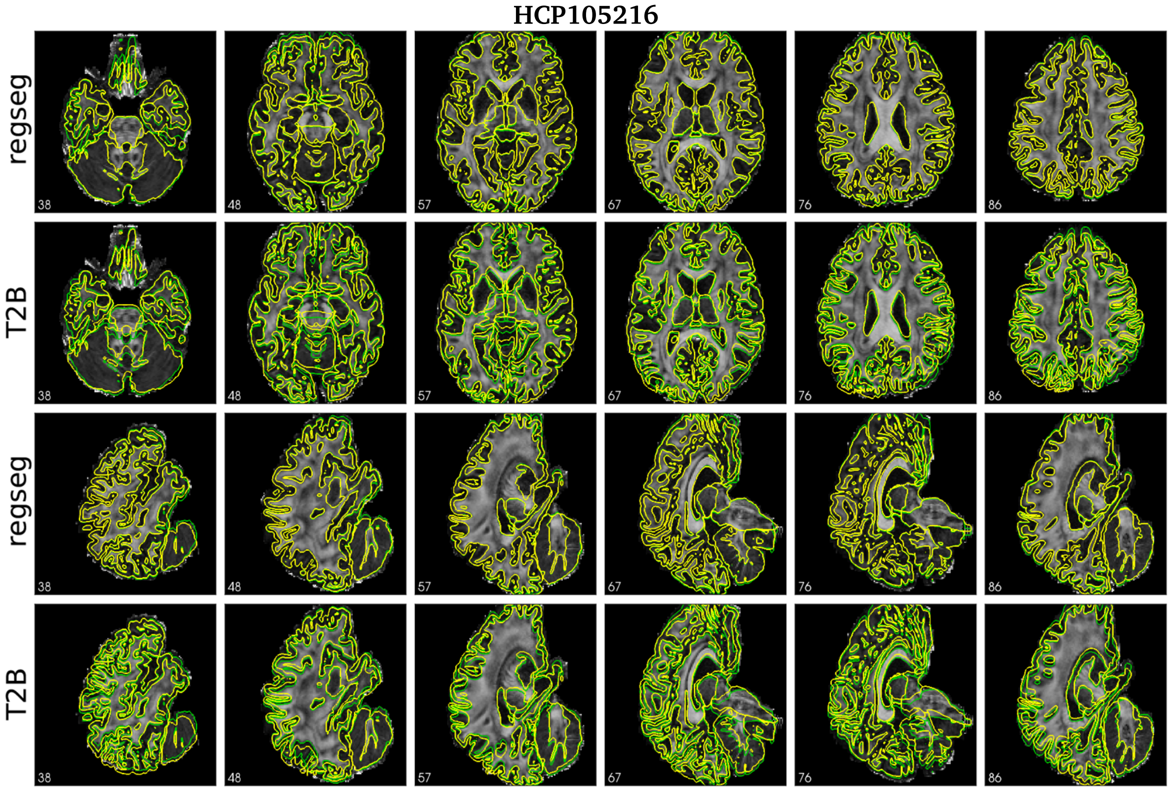

**HCP128127**

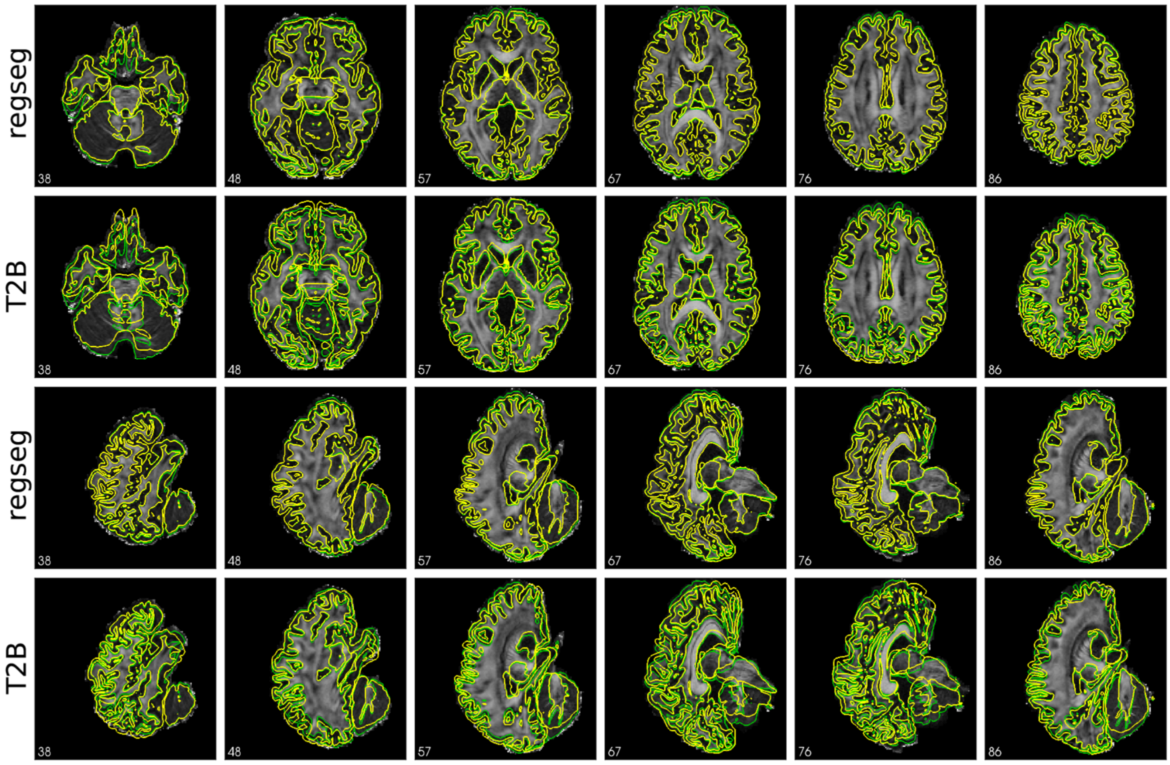


**HCP143325**

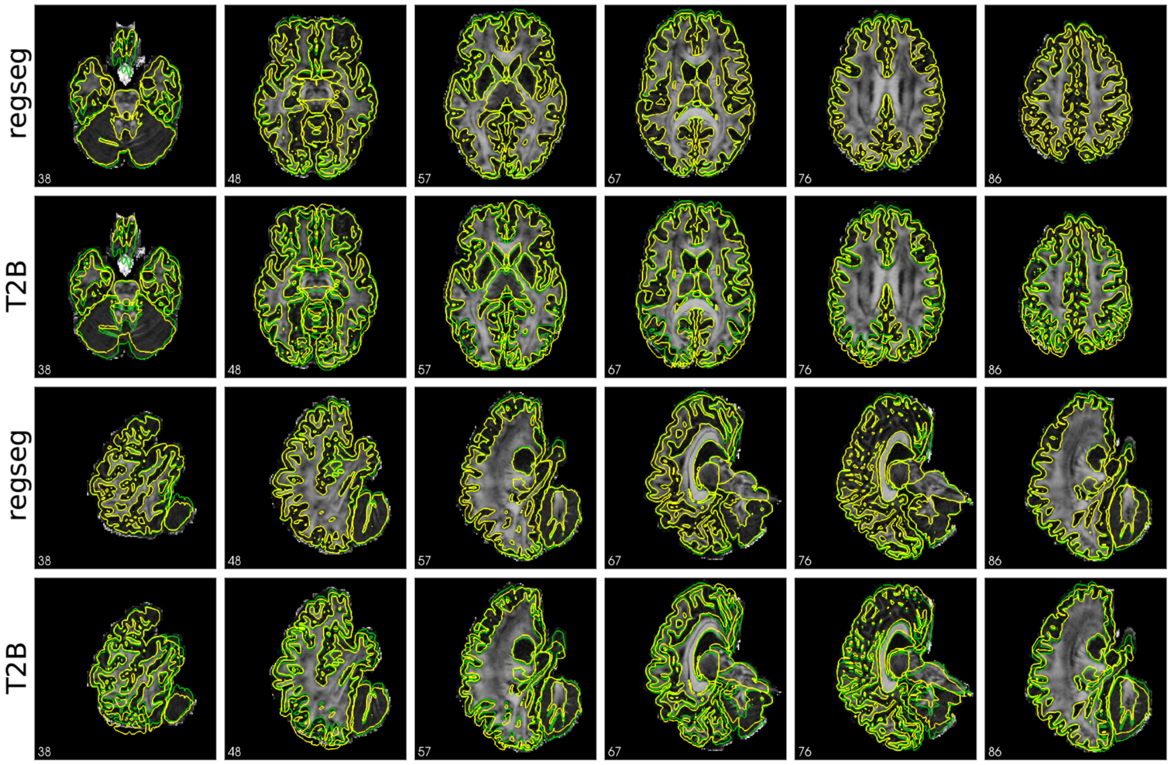

**HCP159441**

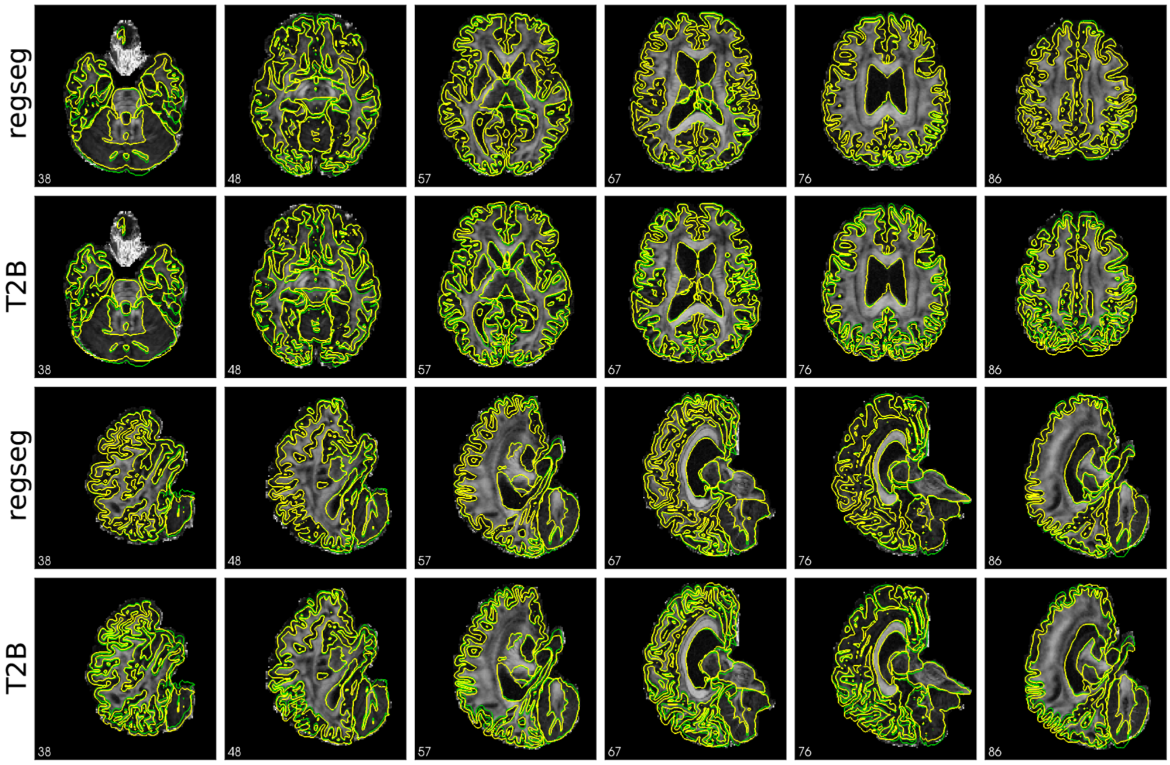


**HCP188347**

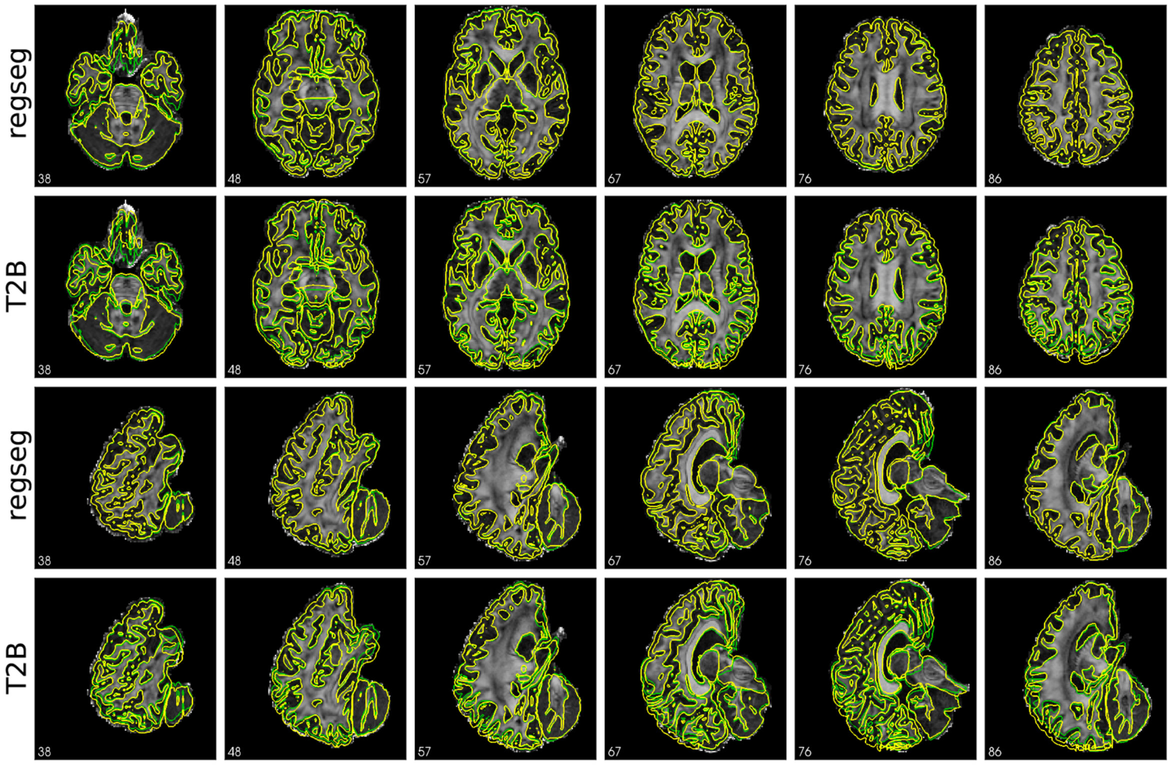

**HCP201818**

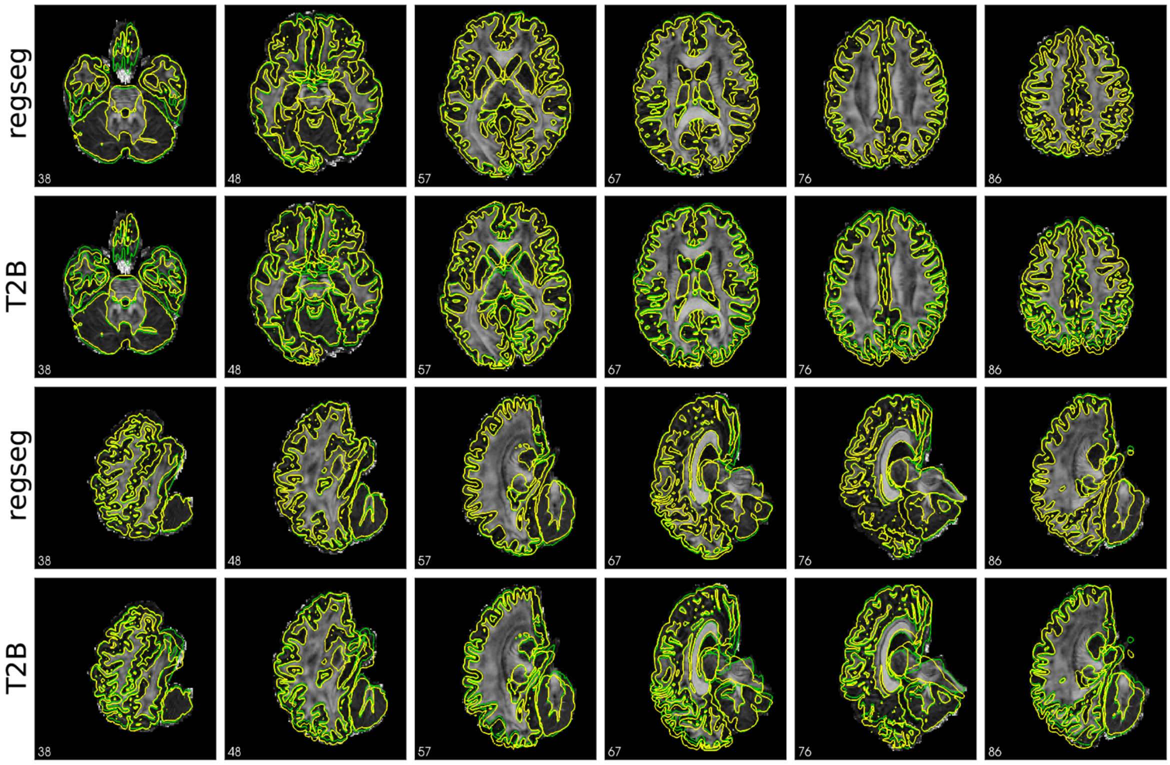


**HCP205220**

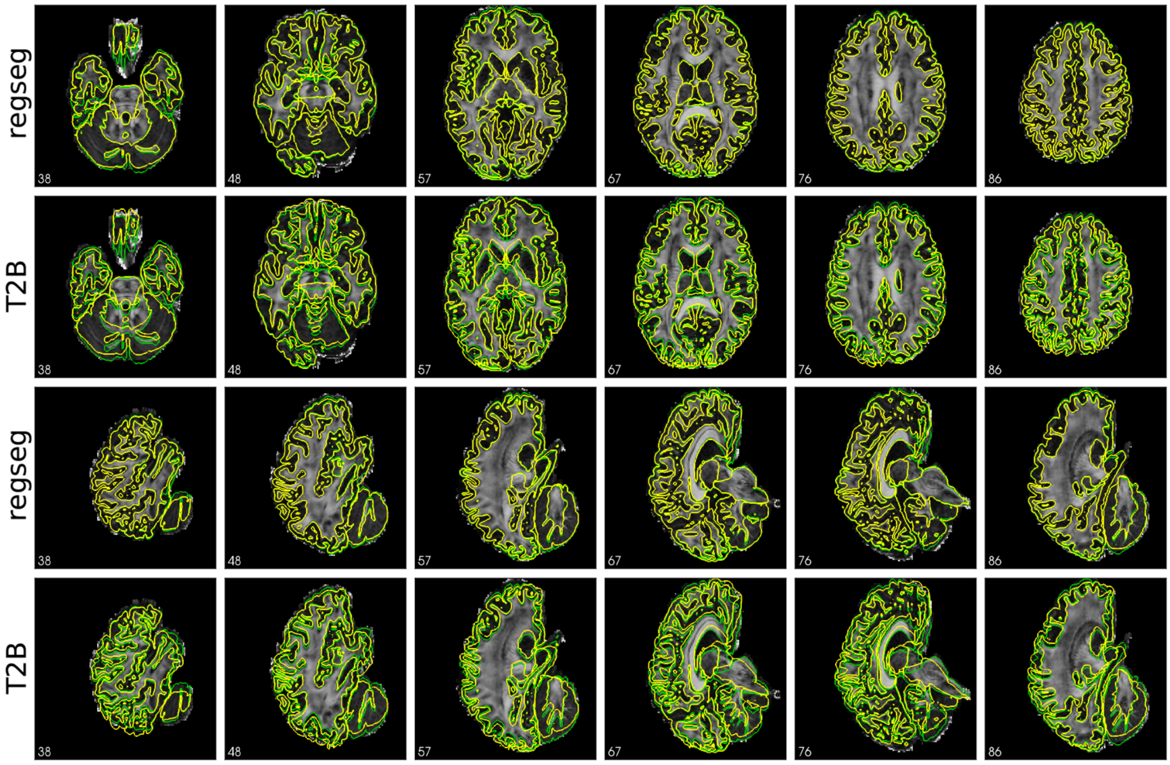

HCP239944

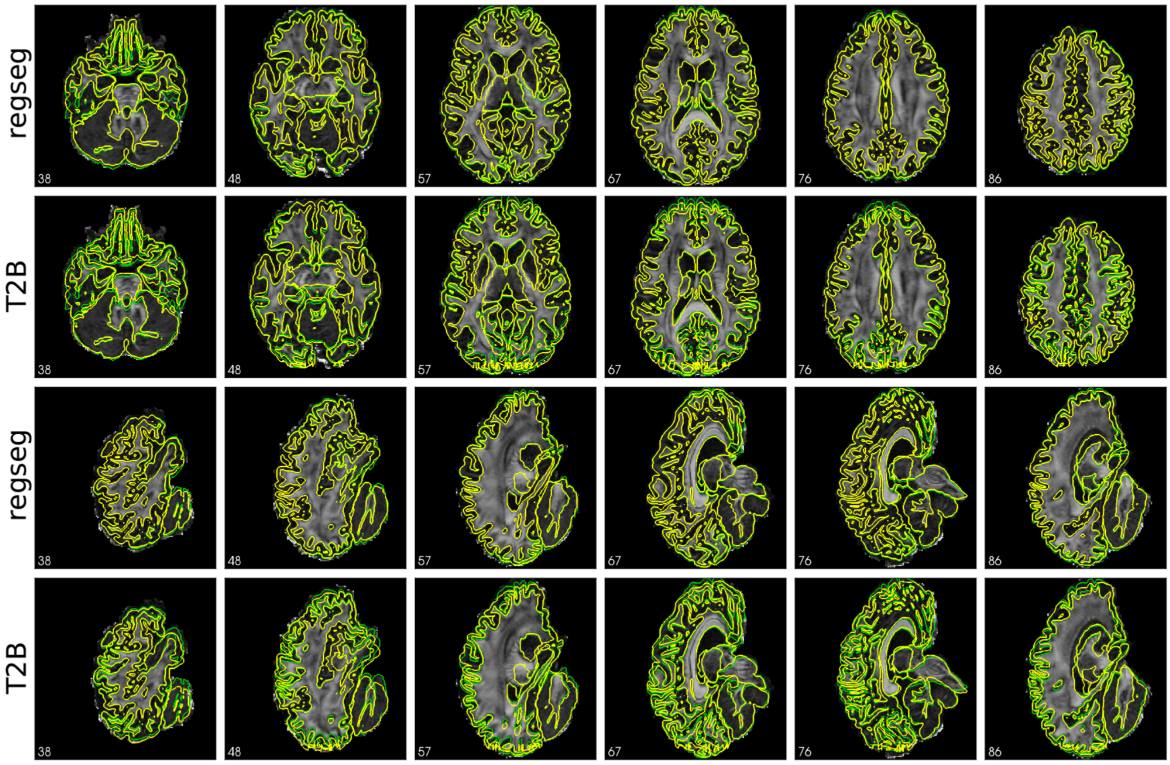


HCP366446

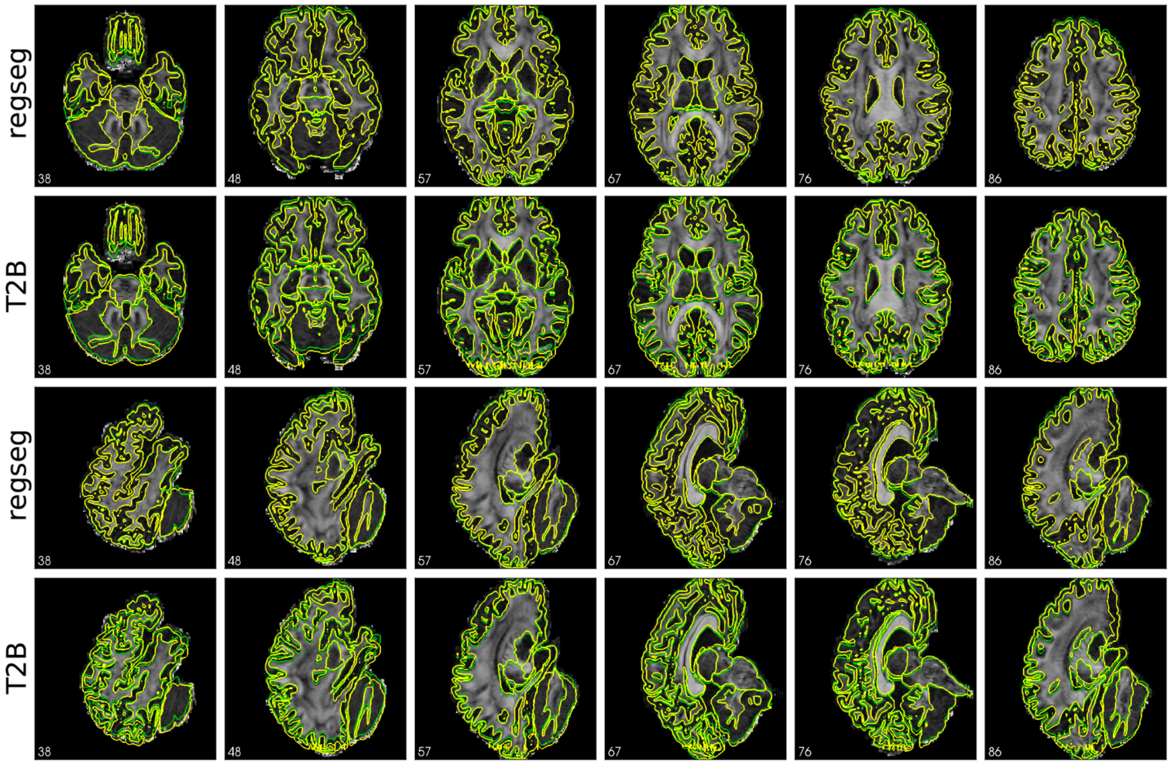

HCP377451

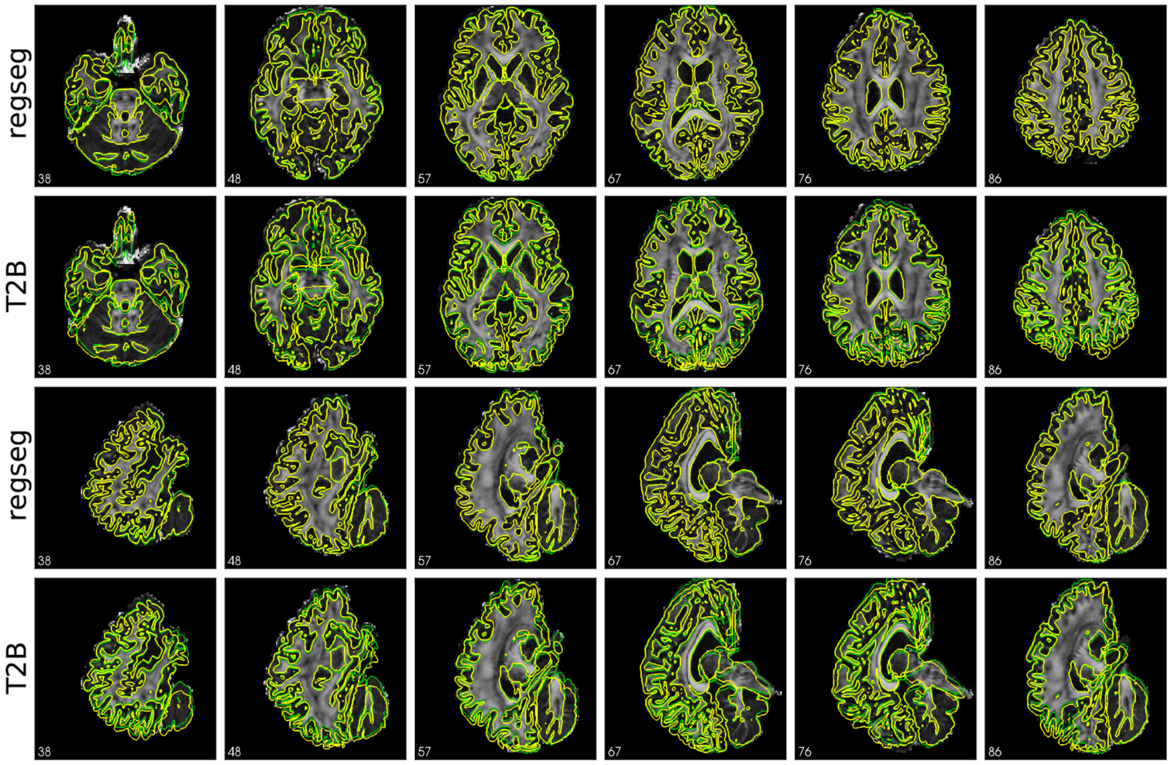


HCP559053

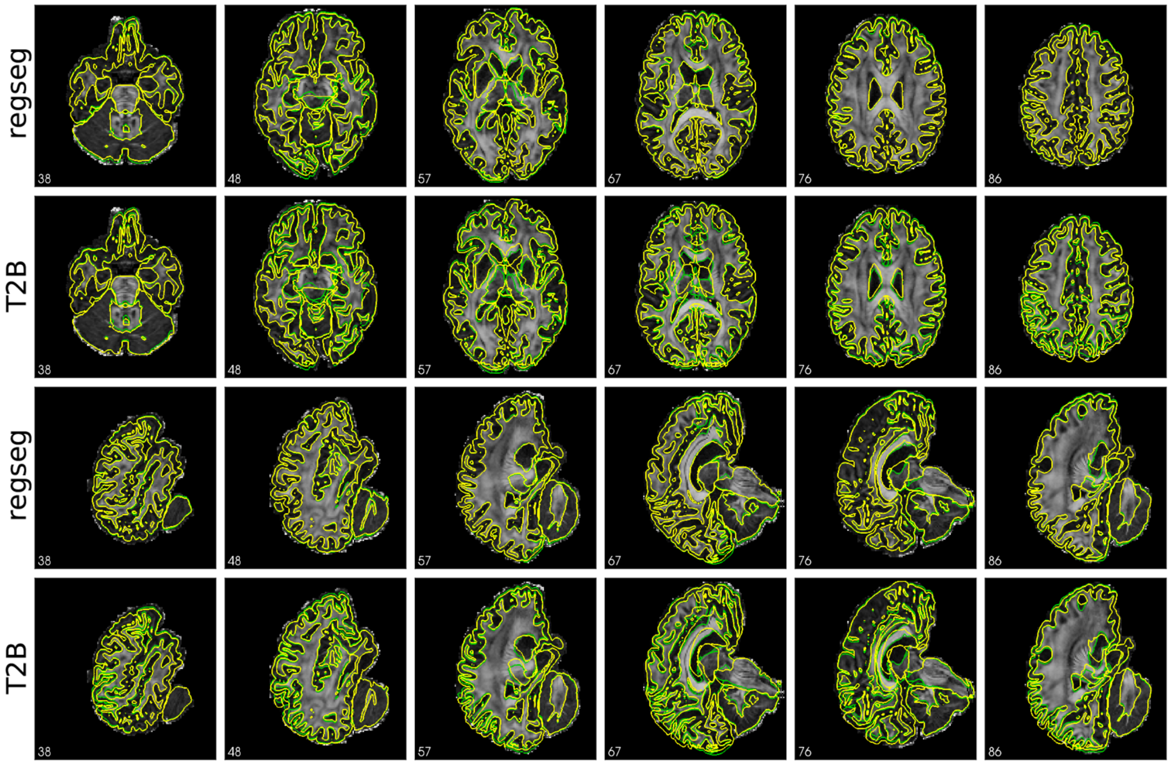

**HCP644044**

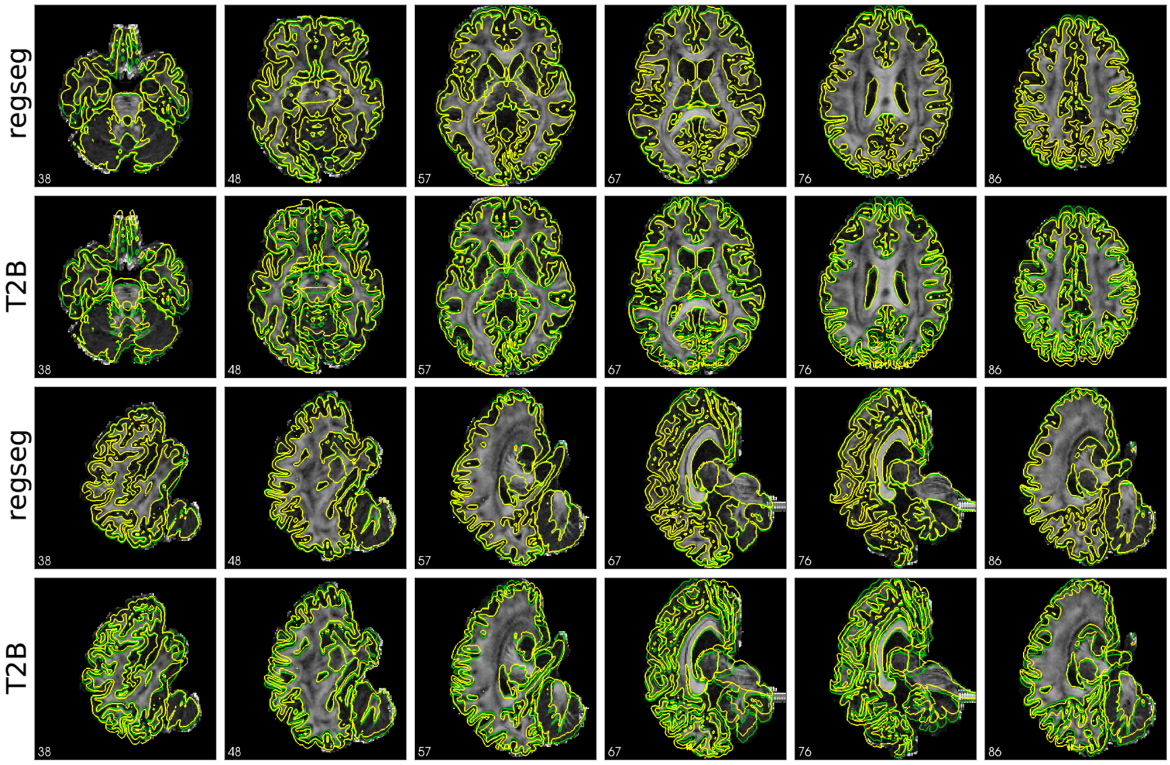


**HCP704238**

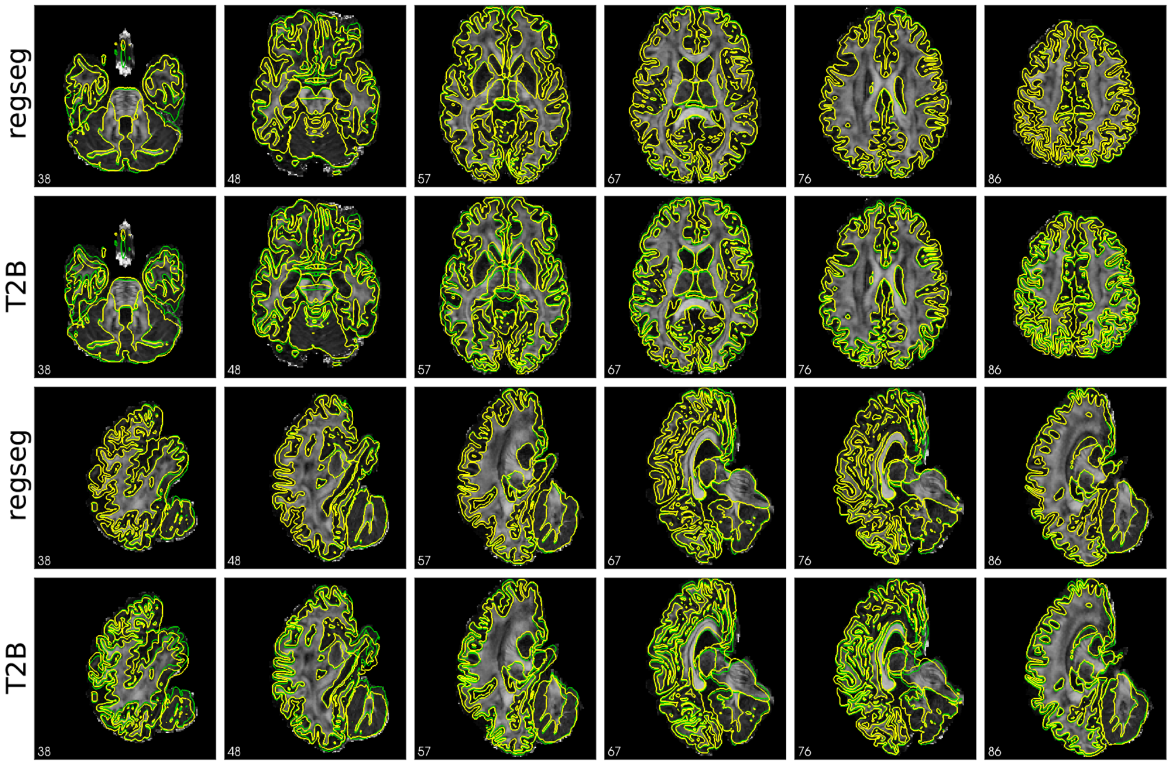

**HCP765056**

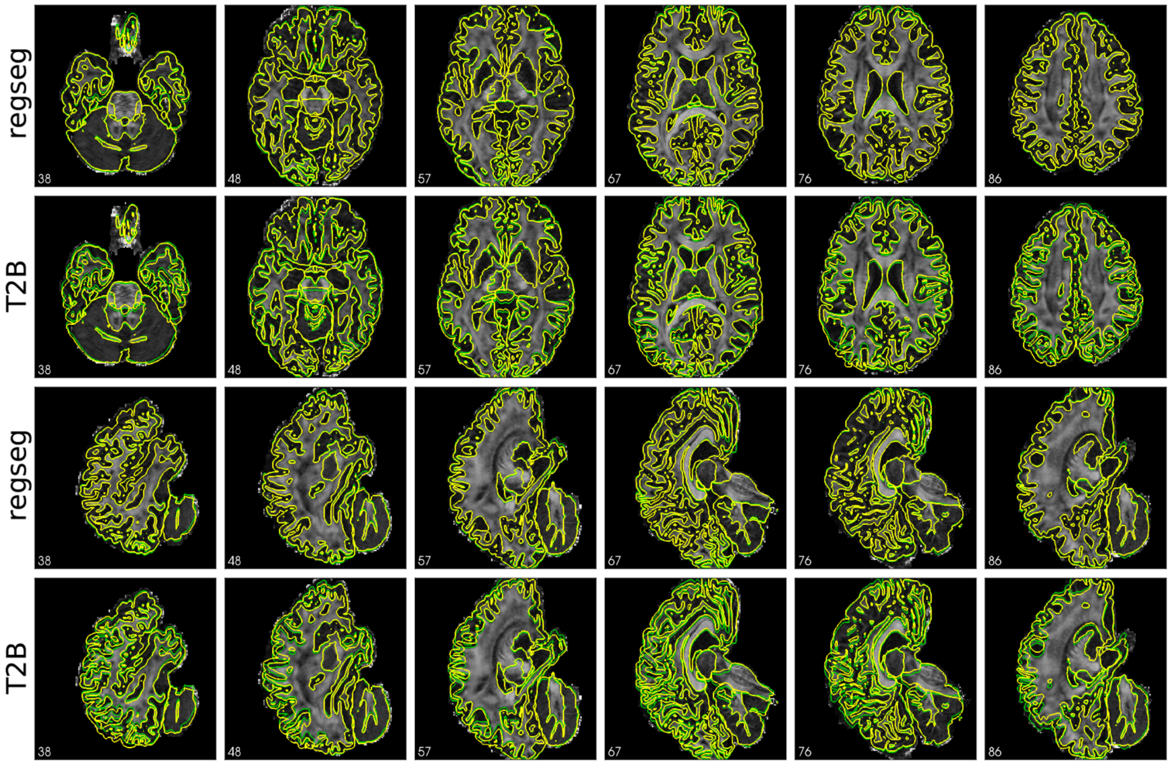


**HCP792564**

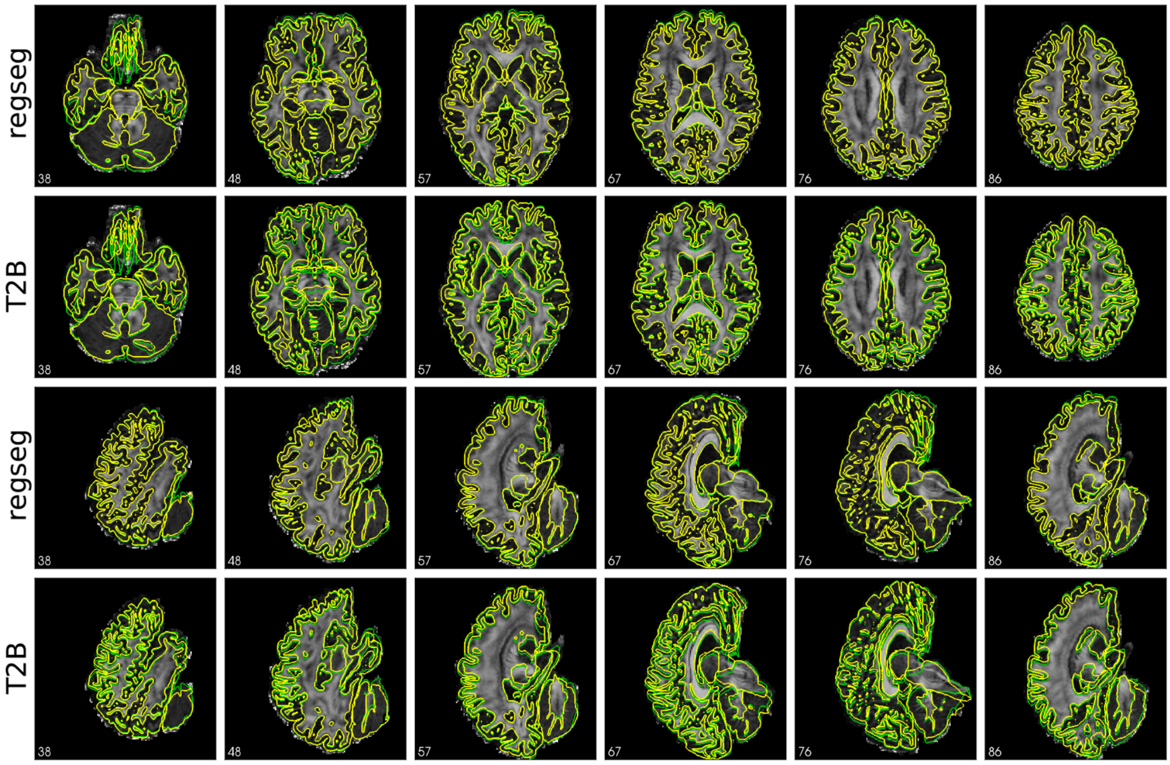

## HCP991267

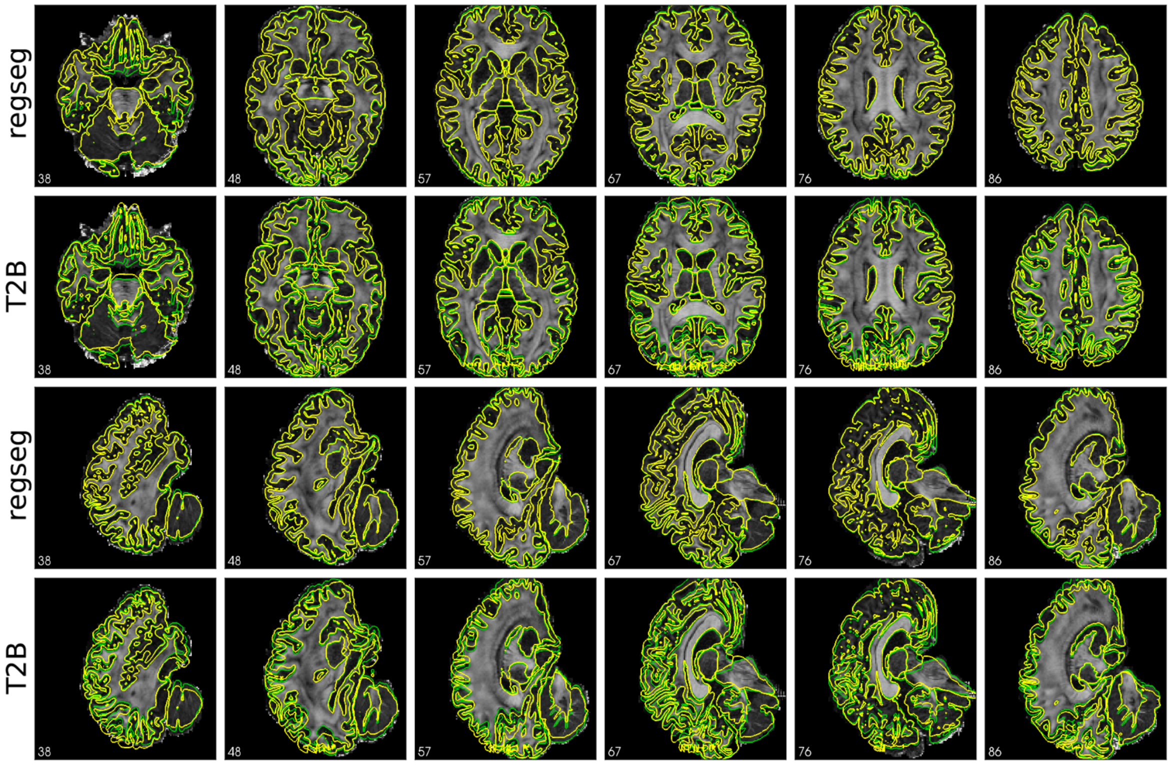

## Author Contributions

This report has been drafted and prepared by OE. Mathematical formulations in section S1 were originally devised and drafted by DZ and rewritten for this document by OE. All the authors have read and critically contributed to this document.

## References


O. Esteban and D. Zosso. RegSeg: Structure-informed segmentation and registration of brain MR images. *Zenodo [Software]*, 2015. doi:10.5281/zenodo.33282.

V. Estellers, D. Zosso, R. Lai, S. Osher, J. Thiran, and X. Bresson. Efficient Algorithm for Level Set Method Preserving Distance Function. *IEEE Trans Image Process*, 21(12):4722–4734, 2012. doi:10.1109/TIP.2012.2202674.

K. Gorgolewski, C. D. Burns, C. Madison, D. Clark, Y. O. Halchenko, M. L. Waskom, and S. Ghosh. Nipype: a flexible, lightweight and extensible neuroimaging data processing framework in Python. *Front Neuroinform*, 5: 13, 2011. doi:10.3389/fninf.2011.00013.

A. Herbulot, S. Jehan-Besson, S. Duffner, M. Barlaud, and G. Aubert. Segmentation of Vectorial Image Features Using Shape Gradients and Information Measures. *J Math Imaging Vis*, 25(3):365–386, 2006. doi:10.1007/s10851-006-6898-y.

L. Ibanez, W. Schroeder, L. Ng, and J. Cates. *The ITK Software Guide*. http://www.itk.org/ItkSoftwareGuide.pdf, second edition, 2005.

J.-D. Tournier, F. Calamante, and A. Connelly. MRtrix: Diffusion tractography in crossing fiber regions. *Int J Imag Syst Tech*, 22(1):53–66, 2012. doi:10.1002/ima.22005.
